# Microbiome-Shaped Metastatic Niches in Colorectal Cancer: Organ-Specific Patterns, Immune-Metabolic Mechanisms, and Therapeutic Translation

**DOI:** 10.3390/microorganisms14081649

**Published:** 2026-07-28

**Authors:** Maochen Luo, Zhuotao Lin, Jiaxin Deng, Yun Zhong, Hui Wang, Qin Liu, Keli Yang

**Affiliations:** 1 Department of General Surgery (Colorectal Surgery), the Sixth Affiliated Hospital, Sun Yat-sen University, Guangzhou 510655, China; luomch6@mail2.sysu.edu.cn (M.L.); linzht6@mail2.sysu.edu.cn (Z.L.); dengjx23@mail3.sysu.edu.cn (J.D.); zhongy326@mail2.sysu.edu.cn (Y.Z.); wang89@mail.sysu.edu.cn (H.W.); 2 Guangdong Provincial Key Laboratory of Colorectal and Pelvic Floor Diseases, the Sixth Affiliated Hospital, Sun Yat-sen University, Guangzhou 510655, China; 3 Key Laboratory of Human Microbiome and Chronic Diseases, Sun Yat-sen University, Ministry of Education, Guangzhou 510655, China; 4 Biomedical Innovation Center, the Sixth Affiliated Hospital, Sun Yat-sen University, Guangzhou 510655, China; 5 Centre for Chinese Herbal Medicine Drug Development, Hong Kong Baptist University, Hong Kong SAR, China

**Keywords:** metastatic colorectal cancer, gut microbiome, tumor microenvironment, biomarkers, therapeutic resistance

## Abstract

Despite advances in systemic therapies, metastatic colorectal cancer (mCRC) remains largely incurable, underscoring persistent gaps in our understanding of metastatic progression and therapeutic resistance. Emerging evidence suggests that gut and tumor-associated microbial communities may contribute to metastatic progression by shaping organ-specific niches, disrupting intestinal and vascular barriers, remodeling immune and stromal microenvironments, and altering host–microbial metabolism. This review synthesizes current evidence on the involvement of gut and intratumoral microbial communities in colorectal cancer metastasis, with emphasis on liver, lung, lymphatic, and peritoneal metastatic patterns; microbial translocation and barrier dysfunction; microbiome–tumor microenvironment interactions; and metabolic pathways such as bile acid, short-chain fatty acid, and tryptophan metabolism. Furthermore, distinct microbial signatures have been associated with responses to chemotherapy, radiotherapy, immunotherapy, and targeted therapies, supporting their potential value as candidate biomarkers for treatment stratification and prognosis, particularly when interpreted alongside treatment exposure and longitudinal microbiome dynamics. Finally, we discuss microbiome-targeted interventions as emerging adjunctive strategies that may help modulate treatment response, while emphasizing the need for standardized, longitudinal, and mechanistically validated studies before clinical translation in mCRC.

## 1. Introduction

Colorectal cancer (CRC) represents a substantial global health challenge, ranking as the third most common malignancy and the second leading cause of cancer-related mortality worldwide. In 2022, CRC accounted for approximately 1.93 million new cases and 904,000 deaths globally [1]. Despite advances in screening and detection, 20% of patients still present with metastatic disease at initial diagnosis, and many others subsequently develop distant relapse [2,3]. The liver, lungs, and peritoneum are the most common metastatic sites. Prognosis is particularly poor for distant-stage CRC, with a 5-year relative survival of approximately 15%, compared with more than 91% for localized disease [4]. Although modern systemic therapies have improved survival in selected patients, metastatic CRC (mCRC) remains largely incurable in most cases. Thus, there is an urgent need to better understand biological factors associated with metastatic progression and therapeutic resistance, and to identify clinically actionable mechanisms that may improve current treatment strategies.

CRC progression has traditionally been attributed to genetic, epigenetic, and environmental factors; however, accumulating evidence also implicates gut microbiome dysbiosis as an important contributor to tumor progression and metastasis. Recent studies have linked the gut microbiome to metastatic CRC across distinct organ sites, including the liver, lungs, and peritoneum [5,6,7,8]. Among the bacterial taxa most frequently investigated in relation to CRC metastasis, *Fusobacterium nucleatum* (*F. nucleatum*), *Escherichia coli* (*E. coli*), and enterotoxigenic *Bacteroides fragilis* (*ETBF*) have received particular attention. In selected experimental and clinical contexts, these taxa have been linked to epithelial–mesenchymal transition, epithelial barrier dysfunction, tumor microenvironment remodeling, and altered antitumor immunity. Beyond bacterial dysbiosis, emerging multi-kingdom analyses suggest that the gut virome and mycobiome may also participate in immune and metabolic remodeling, although their specific roles in metastatic CRC remain less well defined than those of bacterial communities [9,10,11,12]. Together, these findings highlight the broader relevance of the gut microbiome in the biology of metastatic CRC and raise the possibility that microbial communities may also influence treatment response.

Beyond metastatic progression, the microbiome has been increasingly implicated in therapeutic response in mCRC. Clinical and translational studies suggest that microbial composition and dysbiosis may shape the efficacy and toxicity of systemic therapies, including chemotherapy, targeted therapy, and immunotherapy, and may also be associated with radiotherapy response [13]. These observations have prompted growing interest in microbiome-targeted strategies as adjuncts to current mCRC treatment.

Despite growing recognition of the gut and tumor-associated microbiome in mCRC, current evidence remains fragmented and is still largely focused on selected bacterial taxa. Previous reviews have largely addressed the CRC microbiome in relation to tumorigenesis, general immune modulation, or treatment response, whereas fewer have integrated microbial mechanisms with organ-specific metastatic niches and clinical translation in mCRC. The strength of the present review is its organ-specific and mechanism-oriented framework. We synthesize evidence linking gut and intratumoral microbial communities to liver, lung, lymphatic, and peritoneal metastatic patterns, and we connect these observations with barrier dysfunction, microbial translocation, immune and stromal microenvironment remodeling, extracellular matrix remodeling, and host–microbial metabolic pathways. We also evaluate the potential of microbial signatures as candidate biomarkers and discuss microbiome-based interventions across chemotherapy, radiotherapy, immunotherapy, and targeted therapy. Importantly, we further highlight key unresolved issues, including causality versus association, taxon-level overgeneralization, low-biomass contamination, ecological selection, treatment-period microbiome dynamics, and cross-study reproducibility. By integrating organ-specific metastatic biology, immune–metabolic and stromal mechanisms, biomarker development, therapeutic translation, and methodological limitations, this review aims to provide a more comprehensive and clinically oriented framework for understanding microbiome-associated mCRC.

## 2. Microbiome and Its Relationship with Different Metastatic Sites

### 2.1. Gut–Liver Axis and Hepatic Immune Remodeling

**Figure 1:**
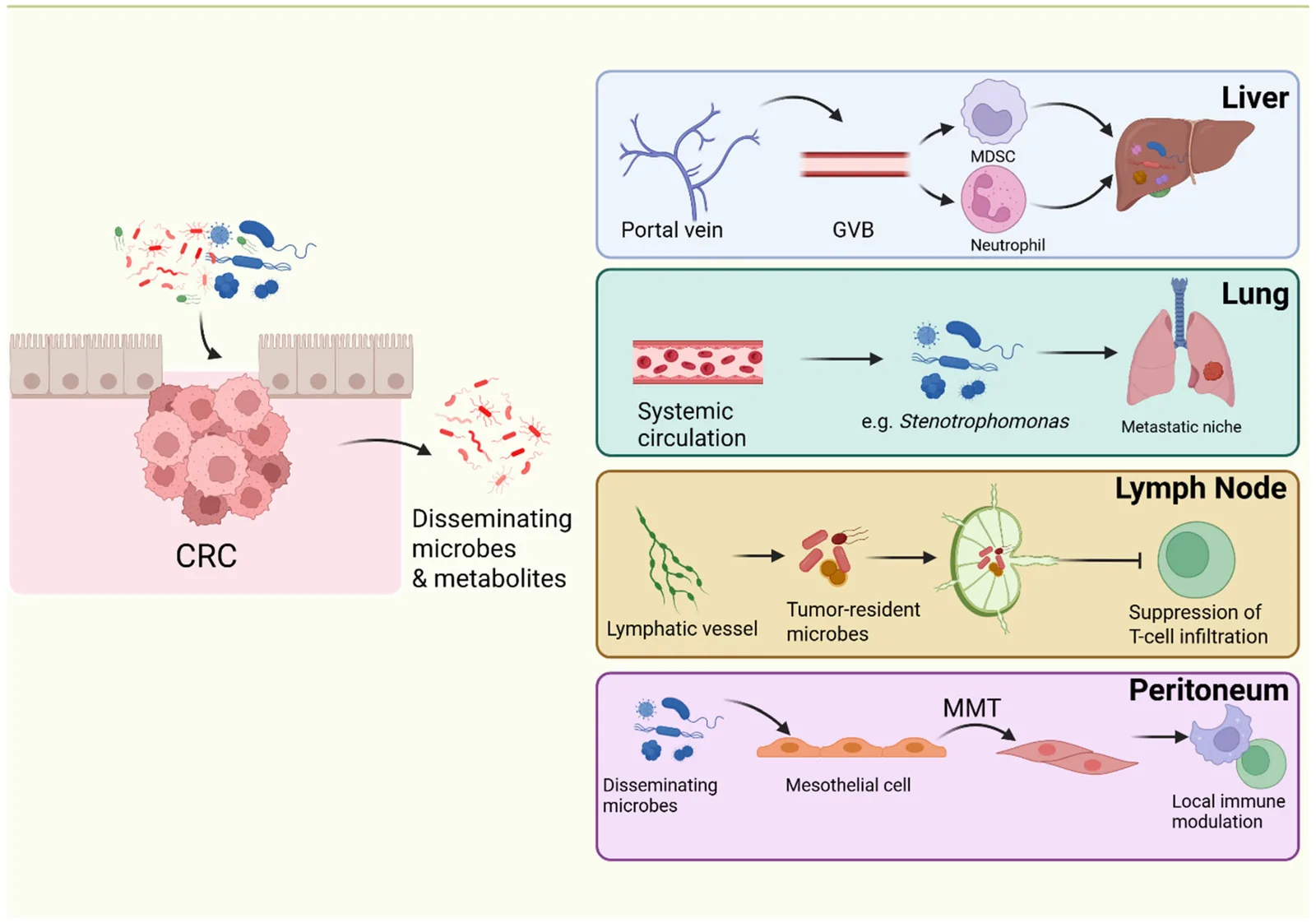
Organ-specific microbiome-associated patterns and proposed mechanisms in metastatic colorectal cancer. This figure summarizes proposed microbiome-associated mechanisms across major metastatic sites in colorectal cancer (CRC). In the liver, intestinal and gut–vascular barrier (GVB) disruption may increase portal exposure to microbial signals, promote myeloid-derived suppressor cell (MDSC) recruitment, and contribute to hepatic immune remodeling. In the lung, circulating microbial products and outer membrane vesicles may influence pulmonary immune reprogramming, although CRC-specific evidence remains limited. In lymphatic dissemination, microbial enrichment may be associated with T-cell exclusion, neutrophil recruitment, and nodal involvement. In the peritoneum, microbial dysbiosis may interact with mesothelial injury, mesothelial-to-mesenchymal transition (MMT), stromal remodeling, and local immune modulation.

The following subsections discuss the organ-specific microbiome-associated patterns and proposed mechanisms summarized in Figure 1, beginning with the gut–liver axis and colorectal liver metastasis. The liver is the most frequent distant metastatic site in CRC, and colorectal liver metastasis (CRLM) markedly worsens stage and prognosis. Increasing evidence has linked gut and tumor-associated microbiome dysbiosis to CRLM risk, although whether these microbial alterations precede metastatic dissemination or reflect ecological selection within metastatic tissues remains incompletely resolved. For example, 16S rRNA profiling of tumors from patients with and without liver metastases identified three intratumoral microbial subtypes that stratified CRLM risk; a genus-level microbial signature including *Agrobacterium*, *Fusobacterium*, *Methylobacterium*, and *Faecalibacterium* achieved an area under the receiver operating characteristic curve (AUC) of 0.78, indicating moderate discriminatory performance [14]. Comparative studies of tissue-associated microbiome have also reported enrichment of selected taxa, including *B. fragilis*, and depletion of *Streptococcus* in metastatic CRC compared with non-metastatic tumors. These observations suggest that tissue-associated microbial composition may help stratify CRLM risk, while the causal contribution of these taxa to metastatic dissemination requires further validation [15]. Concurrently, tumor colonization by virulence-associated bacteria has been associated with advanced disease stage. *F. nucleatum* is abundant on stage III/IV tumor surfaces and has been reported to invade adjacent normal mucosa [16]. *F. nucleatum* has also been detected in both primary tumors and paired liver metastases, a pattern that is consistent with possible microbial co-migration, metastatic-site colonization, or shared ecological selection across lesions. Bullman et al. cultured viable *Fusobacterium* from CRLM lesions that were genomically concordant with strains in the matched primary tumors, supporting the possibility that selected microbial strains may “hitchhike” during metastatic dissemination [17]. Overall, these findings link microbiome dysbiosis and tumor-associated microbial composition with CRLM occurrence and provide a rationale for further investigation into microbial co-dissemination, metastatic-site colonization, and lesion-specific ecological selection [18].

Microbiota dysbiosis has also been associated with heterogeneity in CRLM occurrence, metastatic propensity, tumor biological behavior, and clinical outcomes [19]. Experimental studies provide mechanistic support for a possible immune-mediated link: in mouse models, oral *F. nucleatum* increased peripheral and hepatic pro-inflammatory cytokines, recruited myeloid-derived suppressor cells (MDSCs), reduced natural killer (NK)- and T helper 17 (Th17)-cell infiltration, increased regulatory T (Treg) cells, and was associated with an increased number of hepatic metastatic nodules [20,21]. Collectively, these findings suggest that microbiota-associated immune remodeling may weaken antitumor surveillance and create a more permissive hepatic niche in experimental CRLM models.

These observations have motivated interest in microbiome modulation as a potential adjunctive strategy for CRLM, although the supporting evidence spans preclinical models, exploratory biomarker analyses, and early-phase clinical studies. In mouse models, Bullman et al. showed that metronidazole treatment targeting *Fusobacterium* reduced tumor burden and attenuated CRLM invasiveness [17]. In the immunotherapy setting, baseline gut microbiome composition has been associated with immune checkpoint inhibitor (ICI) response in mCRC. In a Phase Ib/II trial of regorafenib plus the programmed cell death protein 1 (PD-1) antibody toripalimab, the objective response rate (ORR) was 15.2% overall and 8.7% among patients with liver metastases; microbiome profiling showed that high pretreatment fecal *Fusobacterium* abundance was associated with shorter progression-free survival (PFS; median 2.0 vs. 5.2 months) [22]. In another Phase II study of refractory microsatellite-stable (MSS) mCRC, fecal microbiota transplantation (FMT) combined with tislelizumab and fruquintinib achieved a median PFS of 9.6 months and a disease-control rate of 95%. Exploratory analyses suggested that response patterns differed according to baseline microbial composition, with enrichment of *Proteobacteria* and *Lachnospiraceae* in patients with better responses and *Actinobacteria*-rich profiles, including *Bifidobacterium*, in patients with poorer responses [23]. These early clinical observations support further evaluation of baseline microbial composition as a response-associated feature in CRLM-containing mCRC cohorts and provide a rationale for mechanistic studies linking microbial ecology with antitumor immunity and treatment response.

The intratumoral microbiome may also be relevant to chemotherapy response in CRLM. Gu et al. described a metabolic–epigenetic interaction in which abundant *E. coli* colonizing CRLM lesions produced lactate that could serve as a substrate for retinoic acid-inducible gene I (RIG-I) lactylation in macrophages [24]. In this model, RIG-I lactylation impaired immune-sensing signaling and favored macrophage polarization toward an immunosuppressive M2-like phenotype, thereby attenuating adaptive antitumor immunity and contributing to 5-fluorouracil (5-FU) resistance. The same study further showed that pharmacological inhibition of RIG-I lactylation restored 5-FU sensitivity in experimental CRLM models [24]. These findings provide mechanistic support for the concept that selected tumor-associated microbes may modulate chemotherapy response through immune–metabolic and epigenetic pathways, and they suggest a rationale for exploring microbiome-informed adjunctive strategies in CRLM. The CRLM literature therefore frames the liver as a metastatic site in which portal microbial exposure, hepatic immune conditioning, and therapy-related ecological pressures may intersect with metastatic niche biology. Mechanistically, gut microbiota may influence CRLM through several non-mutually exclusive routes: disruption of the intestinal epithelial barrier and GVB, portal delivery of bacteria or microbial products to the liver, activation of hepatic innate immune signaling, recruitment or polarization of myeloid cells, suppression of NK- and T-cell-mediated antitumor surveillance, and microbial metabolite-driven modulation of the hepatic immune niche. These mechanisms provide a biological framework linking gut dysbiosis to liver-specific metastatic susceptibility, while remaining dependent on host, tumor, treatment, and microbial community context.

### 2.2. Gut-Lung Axis and Remote Pulmonary Immunological Reprogramming

The lungs represent the second most frequent site of CRC dissemination, with pulmonary metastases eventually developing in 10–25% of patients over the disease course. Emerging evidence has linked gut and tumor microbiome dysbiosis to CRC invasion and metastatic spread, although direct mechanistic evidence for a CRC-specific gut–lung metastatic axis remains limited [5,25]. Preliminary clinical studies have associated intestinal and tumor microbial features with the propensity for CRC lung metastasis. Comparative fecal microbiome analyses identified a dysbiotic signature in patients with lung metastases, including enrichment of selected taxa and depletion of taxa commonly associated with short-chain fatty acid production or gut homeostasis. Specifically, *Fusobacterium* was enriched in the lung metastasis group, whereas *Bifidobacterium* was more abundant in non-metastatic patients. Taxa such as *Stenotrophomonas* and *Rhodopseudomonas* were also increased in the gut microbiome of patients with lung metastases. Notably, *Stenotrophomonas* was detected in 27.3% (3/11) of lung metastasis cases but was absent in non-metastatic controls, and its fecal abundance positively correlated with serum carcinoembryonic antigen (CEA), a clinical marker associated with CRC recurrence and metastasis. Collectively, these data support an association between fecal microbial composition and CRC lung metastasis and provide a basis for further studies examining whether these signatures reflect microbial participation in pulmonary niche formation, passive ecological selection, or treatment- and host-related confounding.

Beyond fecal communities, tumor-resident microorganisms have also been associated with CRC lung metastasis. A 16S rRNA sequencing study of tumors from 133 patients with metastatic CRC reported significantly higher tumor microbial diversity in patients with lung metastases than in those without lung metastases, as reflected by greater microbial species richness (*p* = 0.026) [6]. Rather than establishing a direct pro-dissemination role, this finding suggests that primary tumors with lung metastatic involvement may harbor distinct or more diverse microbial ecosystems. Multi-omic metagenomic analyses have further compared microbial composition across primary CRC tumors, matched distant metastases, including lung and liver metastases, and normal tissues. These studies showed that microbial profiles differed significantly across lesion types and could distinguish primary tumors, metastases, and normal tissues [26]. Up to 61 viral types, including phages and human viruses, and numerous bacterial species were detected, suggesting lesion-specific microbial heterogeneity and possible microbial shifts during CRC progression. Epstein–Barr virus (EBV) was detected more frequently in cancerous tissue than in normal mucosa, and 122 bacterial species were detected almost exclusively in primary tumors but not in normal tissue. Together, these observations suggest that tumor-associated microbial profiles may have exploratory value for metastasis risk stratification, while their temporal relationship to pulmonary dissemination and their functional relevance require careful interpretation.

However, although microbiome-associated pre-metastatic niche (PMN) formation has been more extensively investigated in the liver, its relevance to other distant organs, such as the lungs, remains less well defined in CRC. Mechanistic clues can be drawn from other solid tumor models, in which high-fat diet-induced gut dysbiosis was associated with pulmonary PMN formation and increased lung metastatic burden [27]. These findings raise the possibility that systemic microbial signals may influence the pulmonary microenvironment, but direct evidence for CRC-specific gut–lung microbial conditioning remains limited. Bacterial outer membrane vesicles (OMVs) have been proposed as one potential mediator of such long-distance communication [28]. As nanoscale carriers of microbial metabolites and virulence-associated factors, OMVs may cross impaired intestinal or vascular barriers and disseminate to distant organs. In experimental settings, OMVs can interact with local immune cells and have been linked to macrophage polarization through Toll-like receptor (TLR)-dependent signaling, providing a biologically plausible mechanism by which microbial products might contribute to pulmonary immune remodeling [29]. Therefore, the putative OMV-mediated gut–lung signaling axis should be considered an emerging hypothesis in CRC metastasis rather than an established mechanism.

Tumor-resident microbiome features have also been associated with prognosis in CRC lung metastasis. Survival analyses indicated that intratumoral detection of several anaerobic genera, including *Prevotella*, *Fusobacterium*, *Selenomonas*, *Peptostreptococcus*, and *Porphyromonas*, was associated with a higher proportion of overall survival < 24 months, whereas the presence of *Sphingomonas* tended to be associated with longer survival. Multivariate models identified *Selenomonas* as an adverse prognostic factor (hazard ratio ≈ 6.35; *p* < 0.001) in combination with elevated CEA [6]. Overall, these findings suggest that tumor microbial profiles may provide prognostic information, although whether these taxa actively contribute to aggressive tumor behavior or instead mark specific ecological states of advanced lesions remains uncertain. A recent study further reported strain-level associations with prognosis, detecting high abundances of the Gram-negative bacillus *Stenotrophomonas maltophilia* in both primary tumors and paired lung metastases from CRC patients [5]. High intratumoral *S. maltophilia* abundance was associated with increased two-year recurrence and mortality, a lower two-year survival rate, more lymph-node metastases, and more frequent vascular invasion [5]. Accordingly, *S. maltophilia* abundance may represent a candidate microbial marker associated with poor prognosis in CRC lung metastasis. Notably, the study also observed site concordance: within individuals, primary tumors and matched lung metastases showed similar microbial compositions, with strong correlations in *S. maltophilia* abundance when overall levels were high [5]. This pattern is consistent with several non-mutually exclusive possibilities, including microbial co-dissemination with tumor cells, shared ecological selection across lesions, or metastatic-site colonization after tumor seeding. Mechanistically, gut microbiota may influence pulmonary metastatic niches through systemic immune and inflammatory signaling rather than direct local colonization alone. Circulating microbial products, metabolites, and outer membrane vesicles may reach distant organs after barrier disruption and can interact with lung-resident immune cells, including macrophages and other myeloid populations. These signals may contribute to pulmonary immune reprogramming, altered cytokine production, and pre-metastatic niche-like conditioning. However, compared with the gut–liver axis, direct CRC-specific evidence for a gut–lung microbiome mechanism remains limited, and lung-associated microbial signatures should therefore be interpreted as candidate mechanistic clues rather than established causal pathways.

### 2.3. Lymphatic Crosstalk and Microbiome-Immune Interplay

Evidence linking the microbiome to CRC lymph node metastasis (LNM) has expanded in recent years, particularly from predictive modeling and clinicopathological correlation studies. A 2025 study of 147 patients developed a fecal microbiome-based machine-learning model for LNM assessment, identifying taxa that tracked with nodal stage and evaluating the model across multiple analytical platforms [30]. These data suggest that preoperative fecal microbiome profiling may complement conventional clinicopathological assessment of regional nodal risk, rather than define the biological cause of lymphatic spread. Tissue-level studies have further connected tumor and adjacent-mucosa microbial profiles with mesenteric nodal involvement; *Fusobacterium* enrichment in node-positive cases coincided with differences in tumor-infiltrating T-cell features. Together, fecal and tissue microbial signatures may serve as candidate adjuncts for LNM risk stratification and for annotating the immune context of node-positive CRC.

Beyond local microenvironmental correlations, recent studies have begun to connect LNM-related microbial patterns with immune and stromal features of lymphatic dissemination. Earlier taxonomic profiling by Yu et al. reported significant microbial shifts in node-positive patients, including enrichment of *Cecembia* and *Asanoa* [31]. More recent work by Wu et al. provided a functional link between *Bacteroides plebeius* enrichment and a C-X-C motif chemokine ligand 8 (CXCL8)–neutrophil inflammatory axis in node-positive CRC. In this setting, *B. plebeius* enrichment coincided with increased CXCL8 expression and neutrophil infiltration, suggesting that selected gut microbial signals may participate in shaping a pro-inflammatory tumor milieu that is permissive to lymphatic spread [30].

Spatial profiling further adds anatomical resolution to the relationship between microbial communities and the immune landscape in node-positive CRC. In particular, *F. nucleatum* has been reported to localize preferentially at the tumor invasive margin, where microbial enrichment was spatially coupled with T-cell exclusion and mesenteric lymph node involvement [32]. This pattern does not by itself establish that boundary-associated bacteria initiate lymphatic dissemination, but it highlights the invasive front as a potential interface where microbial colonization, stromal organization, and local immune exclusion converge.

By contrast, the roles of the mycobiome and virome in CRC LNM are less clearly defined. Most trans-kingdom studies have focused on primary tumorigenesis rather than nodal spread, and available LNM-related data remain limited. Future studies combining deep sequencing, liquid biopsy approaches, spatial multi-omics, and paired primary–node sampling may help determine whether fungal and viral communities participate in local immune or stromal remodeling, reflect advanced tumor ecology, or interact with bacterial communities during lymphatic progression. Mechanistically, microbiome-associated lymphatic dissemination may involve both local and regional immune remodeling. Microbial enrichment at the tumor invasive margin may coincide with T-cell exclusion and stromal reorganization, whereas selected gut microbial signals may promote CXCL8-mediated neutrophil recruitment and inflammatory remodeling in node-positive CRC. These processes could facilitate lymphatic spread by weakening local immune surveillance, altering tumor–stroma interfaces, and creating a pro-inflammatory milieu permissive to lymphatic involvement. Nevertheless, current evidence remains largely associative, and future spatial, longitudinal, and paired primary–node studies are needed to clarify whether these microbial features actively promote lymphatic dissemination or reflect advanced tumor ecology.

### 2.4. Microbiome-Associated Peritoneal Remodeling and Metastasis

Peritoneal metastasis (PM) in advanced colorectal cancer is relatively common and is generally associated with particularly poor prognosis and limited treatment options. According to the Peritoneal Surface Oncology Group International (PSOGI) consensus, PM occurs in roughly 4–15% of patients at initial diagnosis and in up to 25% at recurrence, with overall survival generally worse than that of several other metastatic patterns [33]. In a cohort of 66 patients with malignant ascites, PM showed enrichment trends for the classes *Clostridia* and *Gammaproteobacteria* in the gut microbiome [34]. Although overall microbial diversity was not significantly altered, the presence of PM coincided with shifts in selected microbial groups, suggesting that peritoneal dissemination may be accompanied by distinct gut microbial configurations rather than a uniform loss of microbial diversity. Mechanistically, gut and peritoneal microbial communities may influence CRC peritoneal metastasis (CRC-PM) through immune, mesothelial, and ecological pathways. Microbial dysbiosis may interact with NLRP3 inflammasome-related neutrophil recruitment, local immune imbalance, mesothelial injury, stromal remodeling, and mesothelial-to-mesenchymal transition-like programs that favor peritoneal implantation. In addition, peritoneal ecological features such as ascites, mucin-rich surfaces, necrotic debris, altered oxygen tension, and nutrient availability may shape microbial persistence or detection within peritoneal lesions. These mechanisms suggest a microbiome–peritoneal ecology–implantation axis, although temporal directionality and causal contribution require further validation in contamination-controlled and spatially resolved studies.

Patient-based multi-omics data have begun to place microbial alterations within the tissue ecology of CRC-PM. Integrated single-cell and microbiome sequencing of paired primary tumors, peritoneal metastases, and adjacent normal tissues from 12 patients identified microbial dysbiosis in CRC-PM specimens [35]. In this cohort, selected genera, including *Fusobacterium* and *Bacteroides*, were increased within the tumor microenvironment. Microbial shifts were accompanied by immune remodeling: primary tumors showed marked neutrophil infiltration, possibly linked to NOD-like receptor family pyrin domain-containing 3 (NLRP3) inflammasome activation, whereas peritoneal metastases contained fewer neutrophils. These paired tissue data suggest that microbial composition, neutrophil dynamics, and mesothelial or stromal remodeling may intersect during peritoneal implantation, but they do not establish whether microbial dysbiosis precedes metastatic seeding, emerges from the peritoneal tumor niche, or reflects treatment- and sampling-related influences. Molecular profiling has also revealed gene- and pathway-level distinctions between CRC-PM and non-PM metastases, supporting the future evaluation of microbial features as part of PM classification and patient stratification frameworks [36]. Because peritoneal and metastatic samples are often low-biomass specimens, microbial signals from these tissues are particularly vulnerable to reagent contamination, cross-sample contamination, batch effects, and sequencing or taxonomic-classification artifacts [37,38,39,40]. Future CRC-PM studies should therefore incorporate standardized sampling of peritoneal fluid, nodules, adjacent tissues, and matched controls; negative extraction controls and reagent blanks; batch-aware sequencing and bioinformatic analyses; and quantitative or spatial validation when possible, especially in cohorts linked to cytoreductive surgery (CRS) with or without hyperthermic intraperitoneal chemotherapy (HIPEC) outcomes.

## 3. Molecular Mechanisms Linking the Microbiome to CRC Metastasis

### 3.1. Microbiome–Tumor Immune Microenvironment Interactions and Therapeutic Resistance

ETBF provides one example of how microbial toxins may intersect with immune remodeling in metastatic CRC. ETBF produces *B. fragilis* toxin (BFT), a metalloprotease that has been linked to tumor microenvironmental changes. ETBF has been detected in a substantial proportion of CRC liver metastases and was accompanied by immune features consistent with tumor escape [41]. Mechanistic studies further suggest that BFT can induce interleukin-6 (IL-6), IL-1β, and IL-23 secretion, promoting monocyte recruitment and macrophage polarization toward immunosuppressive phenotypes that support tumor progression in experimental settings [42].

*Streptococcus gallolyticus*, formerly *Streptococcus bovis*, has also been studied in relation to CRC-associated inflammation. In colon cancer models, *S. gallolyticus* activated β-catenin signaling in epithelial cells, with upregulation of c-Myc and Cyclin D1, and increased inflammatory mediators, including tumor necrosis factor-α (TNF-α), IL-6, IL-1β, and IL-8 [43]. These changes provide a mechanistic basis for linking *S. gallolyticus* colonization with inflammatory myeloid-cell recruitment, including IL-8-mediated chemoattraction [44]. *Peptostreptococcus anaerobius* represents another anaerobic taxon connected to immune suppression in CRC. *P. anaerobius* has been reported to preferentially colonize CRC tissues and recruit myeloid-derived suppressor cells (MDSCs) into tumors [45]. In experimental models, MDSC activation, including increased IL-23 secretion, was connected to epithelial–mesenchymal transition (EMT) programs and reduced treatment sensitivity in colon cancer cells [46].

The accumulating MDSCs secrete interleukin-23, which in turn drives a signal transducer and activator of transcription 3 (STAT3)-mediated expansion of immunosuppressive and invasive tumor cell phenotypes. Likewise, colibactin-producing *E. coli* (pks^+^ *E. coli*) has been investigated as a bacterial genotoxicity model linking microbial DNA damage to immune remodeling in CRC. In one study, colon tumors harboring colibactin-positive *E. coli* showed an immune context enriched for suppressive myeloid cells and T helper 17 (Th17) responses, together with reduced responsiveness to immune checkpoint blockade [47]. Colibactin can alkylate host DNA, inducing interstrand crosslinks and DNA double-strand breaks (DSBs), thereby linking microbial genotoxic stress to genomic instability [48]. In response to this injury, tumor and stromal cells may undergo cellular senescence and develop a senescence-associated secretory phenotype (SASP) [49]. SASP-related cytokines and chemokines can modify the extracellular inflammatory milieu and alter cytotoxic T-cell activation [47]. In mouse CRC models, pks^+^ *E. coli* infection was accompanied by exhausted CD8^+^ T-cell phenotypes and reduced anti-PD-1 efficacy [50]. Together, these studies outline a genotoxin–senescence–immune remodeling pathway through which selected *E. coli* strains may influence immunotherapy response in experimental CRC settings.

**Figure 2:**
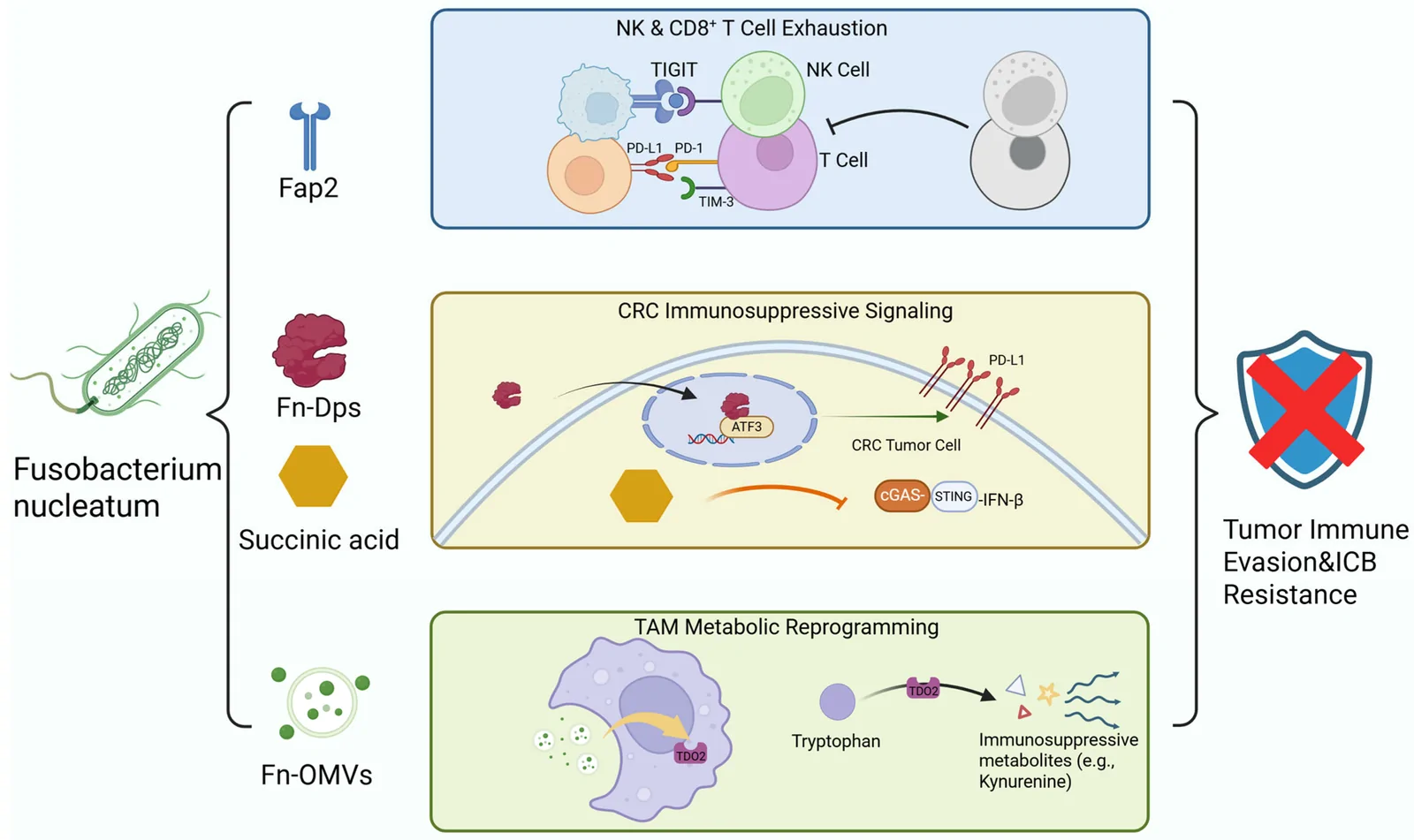
*Fusobacterium nucleatum*-associated immune remodeling and immune checkpoint blockade response in colorectal cancer. *Fusobacterium nucleatum* (*F. nucleatum*) has been linked to immune escape-related pathways in CRC through multiple virulence factors, metabolites, and extracellular vesicles. The bacterial adhesin Fap2 can bind the inhibitory receptor T-cell immunoreceptor with Ig and ITIM domains (TIGIT) on natural killer (NK) cells and T-cells, thereby impairing cytotoxic immune surveillance. Fn-Dps can enter host cells and interact with activating transcription factor 3 (ATF3), leading to increased programmed death-ligand 1 (PD-L1) expression on CRC cells and suppression of antitumor T-cell activity. *F. nucleatum*-derived succinate has been reported to inhibit the cyclic GMP–AMP synthase–stimulator of interferon genes–interferon-β (cGAS–STING–IFN-β) pathway, limiting effector CD8^+^ T-cell recruitment and weakening anti-PD-1 responses in mouse CRC models. In parallel, *F. nucleatum* outer membrane vesicles (Fn-OMVs) can alter tumor-associated macrophage (TAM) metabolism through tryptophan 2,3-dioxygenase 2 (TDO2)-mediated tryptophan metabolism in experimental settings. These pathways provide mechanistic support for a model in which *F. nucleatum* may influence NK-cell activity, CD8^+^ T-cell function, immune escape-related signaling, and responsiveness to immune checkpoint blockade. In the schematic, arrows indicate activation or promotion, blunt-ended lines indicate inhibition or suppression, and the red “X” over the shield represents impaired antitumor immune protection and resistance to immune checkpoint blockade.

*F. nucleatum* represents one of the best-characterized microorganisms linked to immune remodeling and treatment response in CRC [51,52]. Current studies support several complementary mechanisms through which *F. nucleatum* may interfere with antitumor immunity. At the receptor level, the bacterial surface protein Fap2 binds to the inhibitory receptor T-cell immunoreceptor with Ig and immunoreceptor tyrosine-based inhibitory motif (ITIM) domains (TIGIT) on natural killer (NK) cells and T-cells, thereby reducing cytotoxic activity [53]. Intracellularly, the secreted protein Fn-Dps has been reported to enter host cells and interact with activating transcription factor 3 (ATF3), which can increase programmed death-ligand 1 (PD-L1) expression on tumor cells [54]. In parallel, *F. nucleatum*-derived succinate has been shown to suppress the cyclic GMP–AMP synthase–stimulator of interferon genes–interferon-β (cGAS–STING–IFN-β) pathway and limit effector T-cell recruitment [55]. *F. nucleatum*-derived outer membrane vesicles (Fn-OMVs) can also be taken up by tumor-associated macrophages (TAMs), altering tryptophan metabolism through tryptophan 2,3-dioxygenase 2 (TDO2) and contributing to an immune-suppressive macrophage state [51]. Additional studies have linked *F. nucleatum* exposure to impaired NK-cell function [53], tumor-infiltrating CD8^+^ T-cell apoptosis or necrosis [56], and exhausted T-cell phenotypes characterized by increased expression of inhibitory checkpoints such as PD-1, T-cell immunoglobulin and mucin-domain containing-3 (TIM-3), and TIGIT [57]. Taken together, these findings position *F. nucleatum* as a useful experimental model for studying microbiome–immune interactions in CRC. Rather than supporting a single linear pathway, the available evidence points to several convergent immune-regulatory mechanisms through which *F. nucleatum* colonization may weaken antitumor immunity and influence responsiveness to immune checkpoint blockade. These complementary mechanisms are summarized in Figure 2.

In addition to immune remodeling, tumor-associated microbial communities have been linked to chemotherapy response in CRC. *B. fragilis* has been investigated as a contributor to chemoresistance in recent clinical and experimental studies. Clinical cohorts reported enrichment of *B. fragilis* in CRC patients with poor chemotherapy response, together with less favorable survival outcomes. Functional studies further showed that colonization with *B. fragilis* reduced the efficacy of 5-FU and oxaliplatin in tumor-bearing mice [58]. Mechanistically, the *B. fragilis* surface adhesin SusD/RagB can bind Notch1 receptors on cancer cells, activate Notch signaling, and induce epithelial–mesenchymal transition (EMT) and stemness-associated programs, thereby reducing susceptibility to chemotherapy-induced apoptosis. Deletion of SusD/RagB or pharmacological inhibition of Notch signaling reversed this phenotype in experimental models, strengthening the evidence for a specific *B. fragilis*–Notch interaction in chemotherapy response. In the same line of therapeutic exploration, a lytic phage targeting *B. fragilis* selectively depleted the bacterium and enhanced 5-FU response in preclinical CRC models.

Another gut anaerobe, *Peptostreptococcus anaerobius*, has been examined in relation to oxaliplatin response through immune microenvironmental modulation. As noted above, *P. anaerobius* can recruit MDSCs, which may promote EMT programs in tumor cells through MDSC-derived IL-23/STAT3 signaling and thereby reduce oxaliplatin sensitivity in experimental CRC models [46]. In mouse models, *P. anaerobius* colonization was accompanied by a weaker oxaliplatin response, whereas targeting either the bacterium or the IL-23–STAT3 pathway attenuated this resistance phenotype [46]. pks^+^ *E. coli* may add another layer to therapy-associated microbial effects. Beyond the DNA damage and immune effects discussed above, colibactin-producing *E. coli* was reported to promote lipid-droplet accumulation and metabolic reprogramming in tumor cells exposed to therapeutic stress [59]. This lipid-altered state, characterized by increased glycerophospholipids and reduced pro-apoptotic ceramides, was linked to T-cell exhaustion and chemoresistance in right-sided CRC tumors colonized by pks^+^ strains. In experimental systems, removal of the colibactin factor using a mutant *E. coli* strain reversed these metabolic and immune phenotypes, pointing to colibactin-associated metabolic remodeling as a candidate intervention node rather than a fully established clinical target.

Other gut microbes have also been examined in relation to tumor growth and treatment-relevant cellular states. *Enterococcus faecalis*, for instance, can secrete metabolites that engage pro-survival signaling pathways: *E. faecalis*-derived biliverdin was reported to activate phosphoinositide 3-kinase (PI3K)–AKT–mechanistic target of rapamycin (mTOR) signaling in CRC cells and to increase pro-angiogenic and pro-survival factors, including vascular endothelial growth factor A (VEGF-A) and IL-8 [60]. In vitro, *E. faecalis* metabolites accelerated CRC cell proliferation and reduced cell-cycle arrest, whereas in mouse models they were accompanied by increased tumor vascularization and growth [60]. Although these findings are not directly linked to resistance against a specific anticancer drug, they suggest that *E. faecalis*-derived metabolites may shape growth- and angiogenesis-related tumor states that could influence therapeutic sensitivity. Finally, gut bacteria can influence anticancer therapy through drug-metabolizing enzymes and microbial metabolites. For example, bacterial β-glucuronidases can deconjugate the inactive irinotecan metabolite SN-38 glucuronid (SN-38G) back to SN-38 in the intestine, thereby contributing to gastrointestinal toxicity and potentially altering local drug exposure. Selected bacterial taxa have also been reported to metabolize fluoropyrimidines such as 5-FU, although the species-specific contribution and clinical relevance of this process in CRC require careful source-specific interpretation [61]. These examples broaden the therapeutic framework beyond immune modulation by showing that microbial communities may affect treatment through pharmacokinetic, metabolic, and immune-mediated routes.

Accordingly, antibiotics, bacteriophages, enzyme inhibition, and microbiome modulation are being explored as approaches to adjust microbial influences on treatment exposure, toxicity, and response in metastatic CRC [58]. In the chemotherapy setting, evidence from gastrointestinal cancer models has linked *F. nucleatum* exposure to autophagy activation, tumor-cell survival pathways, and DNA damage repair programs, which may reduce drug sensitivity in selected experimental contexts [62,63]. However, extrapolation of these findings to CRC-specific chemotherapy resistance should be made cautiously and supported by CRC-focused studies.

In the immunotherapy setting, *F. nucleatum* has been linked to variable responses to immune checkpoint inhibitors (ICIs), particularly through immune-metabolic mechanisms. In selected studies of anti-PD-1/PD-L1 therapy, higher *F. nucleatum* abundance in tumor tissue or feces was connected with poorer treatment response [55]. Mechanistically, *F. nucleatum* can influence PD-L1 expression and alter cyclic GMP–AMP synthase–stimulator of interferon genes–interferon-β (cGAS–STING–IFN-β) signaling [55]. For example, *F. nucleatum*-derived succinate inhibited the cGAS–STING–IFN-β pathway in tumor cells, limited CD8^+^ T-cell infiltration, and reduced anti-PD-1 activity in mouse CRC models. In these models, depletion of tumor-associated *F. nucleatum* with anaerobic antibiotics lowered serum succinate levels and improved anti-PD-1 responsiveness. Separately, *F. nucleatum*-derived outer membrane vesicle (Fn-OMV)-mediated metabolic reprogramming of tumor-associated macrophages (TAMs) through tryptophan 2,3-dioxygenase 2 (TDO2) was shown to reduce anti-PD-1 efficacy in a head and neck squamous cell carcinoma (HNSCC) model, whereas TDO2 inhibition enhanced treatment response [51]. This body of evidence supports a context-dependent model in which *F. nucleatum*-related metabolites and vesicles may influence ICI responsiveness through T-cell recruitment, macrophage metabolism, and checkpoint-regulatory pathways, without establishing *F. nucleatum* as a universal marker of immunotherapy resistance.

### 3.2. Microbiome–Stromal Matrix Remodeling and Biophysical Niche Formation

In addition to immune-cell remodeling, microbial communities and microbial products may influence the stromal and biophysical architecture of the tumor microenvironment (TME). The cancer microbiome has been increasingly recognized as part of the broader tumor ecosystem, with potential roles in immune modulation, epithelial signaling, and treatment response [64]. In CRC, microbial organization itself may alter tissue biology: bacterial biofilms have been associated with epithelial invasion, reduced E-cadherin expression, increased IL-6/STAT3 signaling, and enhanced epithelial proliferation, suggesting a possible link between microbial spatial organization, barrier disruption, and stromal exposure to microbial products [65].

Several molecular routes may connect microbiome-associated inflammation with stromal and extracellular matrix (ECM) remodeling. Tumor-associated bacteria, bacterial toxins, pathogen-associated molecular patterns, and microbial metabolites can activate epithelial cells, macrophages, mesothelial cells, and cancer-associated fibroblasts (CAFs), thereby altering cytokine release, fibroblast activation, ECM deposition, and matrix-degrading enzyme activity. For example, *F. nucleatum* binding and invasion of CRC cells can induce IL-8 and C-X-C motif chemokine ligand 1 (CXCL1) secretion and promote cancer-cell migration, while another study reported that *F. nucleatum* upregulated matrix metalloproteinase 7 (MMP7) through the mitogen-activated protein kinase (MAPK)–c-Jun N-terminal kinase (JNK)–activator protein 1 (AP-1) axis to enhance metastasis-related CRC phenotypes [66,67]. More broadly, CAFs are central regulators of ECM structure, matrix deposition and remodeling, metabolic reprogramming, and immune crosstalk within the TME [68,69].

From a biophysical perspective, ECM remodeling may create a permissive niche for invasion and metastatic colonization by changing collagen organization, basement-membrane integrity, tissue stiffness, interstitial pressure, vascular permeability, and immune-cell trafficking. The tumor-associated ECM is not merely a scaffold; it provides biochemical and biomechanical cues that influence cancer-cell growth, survival, migration, angiogenesis, immune function, and metastatic dissemination [70,71,72]. Matrix metalloproteinases (MMPs) may further promote invasion and metastasis by degrading structural ECM components and modifying growth factors, adhesion molecules, and other signaling substrates [73]. Conversely, remodeled matrix architecture may also shape microbial localization by generating hypoxic, necrotic, mucin-rich, or poorly perfused microhabitats that favor the persistence or detection of selected taxa. Thus, microbiome–TME remodeling should be viewed as bidirectional: microbial signals may modulate stromal and matrix architecture, whereas the remodeled ECM and biophysical gradients may select for particular microbial communities.

### 3.3. Gut–Vascular Barrier Disruption and Microbial Translocation

In solid tumors such as CRC, disruption of the intestinal barrier may facilitate the translocation of microbes and microbial products into the systemic and portal circulation, thereby exposing distant organs to microbial signals that can influence local immune and stromal states. The intestinal barrier comprises two coordinated lines of defense: the mucosal epithelial barrier and the gut–vascular barrier (GVB) [74]. Intact epithelial tight junctions limit microbial access to tissue [50,59], whereas the submucosal vascular endothelium further constrains hematogenous bacterial dissemination [43,75]. The portal vein provides a major anatomical route linking the intestine and the liver. Under physiological conditions, venous blood from the intestine first enters the liver through the portal vein, where Kupffer cells and other hepatic immune cells clear low-level microbial products that reach the portal circulation and limit microbial exposure within the liver [76,77]. When primary tumors, inflammation, or other host factors compromise epithelial barrier integrity, bacteria and microbial products may cross the mucosa and enter deeper tissue compartments. If the GVB is also impaired, microbial components may pass through compromised submucosal endothelial barriers and reach the portal circulation, thereby increasing microbial exposure in the liver [78]. Increased expression of the endothelial permeability protein plasmalemma vesicle-associated protein 1 (PV-1) has been used as a marker of GVB disruption [75]. In CRC patients, high PV-1 expression in tumor-associated endothelial cells was correlated with postoperative distant metastasis, with the high-PV-1 group showing approximately twice the recurrence or metastasis risk of the low-PV-1 group [78]. In patients with high PV-1 expression, bacterial signals were also detected in liver metastases, often near SOX9^+^ proliferative cancer cells. This spatial pattern links GVB impairment, portal microbial exposure, and the microbial ecology of liver metastases, but it should not be taken as evidence that bacterial colonization necessarily precedes metastatic seeding.

Experimental evidence suggests that bacterial translocation through the portal route can influence the hepatic immune microenvironment and may contribute to PMN-like changes under selected conditions [78]. This process should not be viewed solely as passive leakage caused by tumor-associated inflammation or barrier disruption, because bacterial virulence programs may also participate in GVB impairment. Bertocchi et al. showed that tumor-resident *E. coli* can use the virulence transcription factor VirF to activate the Type III secretion system (T3SS) [78]. Through this secretory apparatus, *E. coli* impaired submucosal endothelial junctions, increased expression of the permeability marker PV-1, and promoted GVB dysfunction in experimental models. This barrier alteration increased portal bacterial translocation and hepatic exposure to *E. coli* and other commensal bacteria. In the liver, disseminated bacterial signals were linked to activation of innate immune sensing and recruitment of myeloid-derived inflammatory cells, creating a hepatic microenvironment more permissive to metastatic seeding in the studied models.

Translocated bacteria and microbial products may activate hepatic innate immune signaling and promote the recruitment of neutrophils, inflammatory monocytes, macrophages, and other bone marrow-derived cells. In experimental models, these immune changes have been associated with a hepatic microenvironment that is more permissive to tumor-cell seeding and survival. Livers with higher bacterial burden showed greater susceptibility to tumor-cell implantation and macroscopic metastatic lesion formation. Barrier disruption may therefore increase portal exposure to bacteria and microbial products, accompanied by changes in endothelial permeability, PV-1 expression, and recruitment or polarization of innate immune cells such as neutrophils and macrophages [78]. These events can support a hepatic microenvironment that is more permissive to metastatic seeding in model systems. From a translational perspective, strategies aimed at preserving intestinal and GVB integrity or limiting bacterial translocation may provide a rationale for reducing metastasis-associated hepatic immune remodeling, but their preventive value for CRLM still needs to be tested in clinically relevant settings.

A clearer understanding of microbiome-related immune, barrier, and metabolic mechanisms may help refine current models of CRC metastatic progression. These mechanistic insights have also motivated early translational efforts to modulate the gut and tumor-associated microbiome through dietary, pharmacological, microbial, or ecosystem-level interventions. Such approaches remain at different stages of preclinical and clinical evaluation and should be interpreted according to the strength of evidence supporting each intervention strategy.

### 3.4. Metabolic Reprogramming via Microbial Metabolite Axes

**Figure 3:**
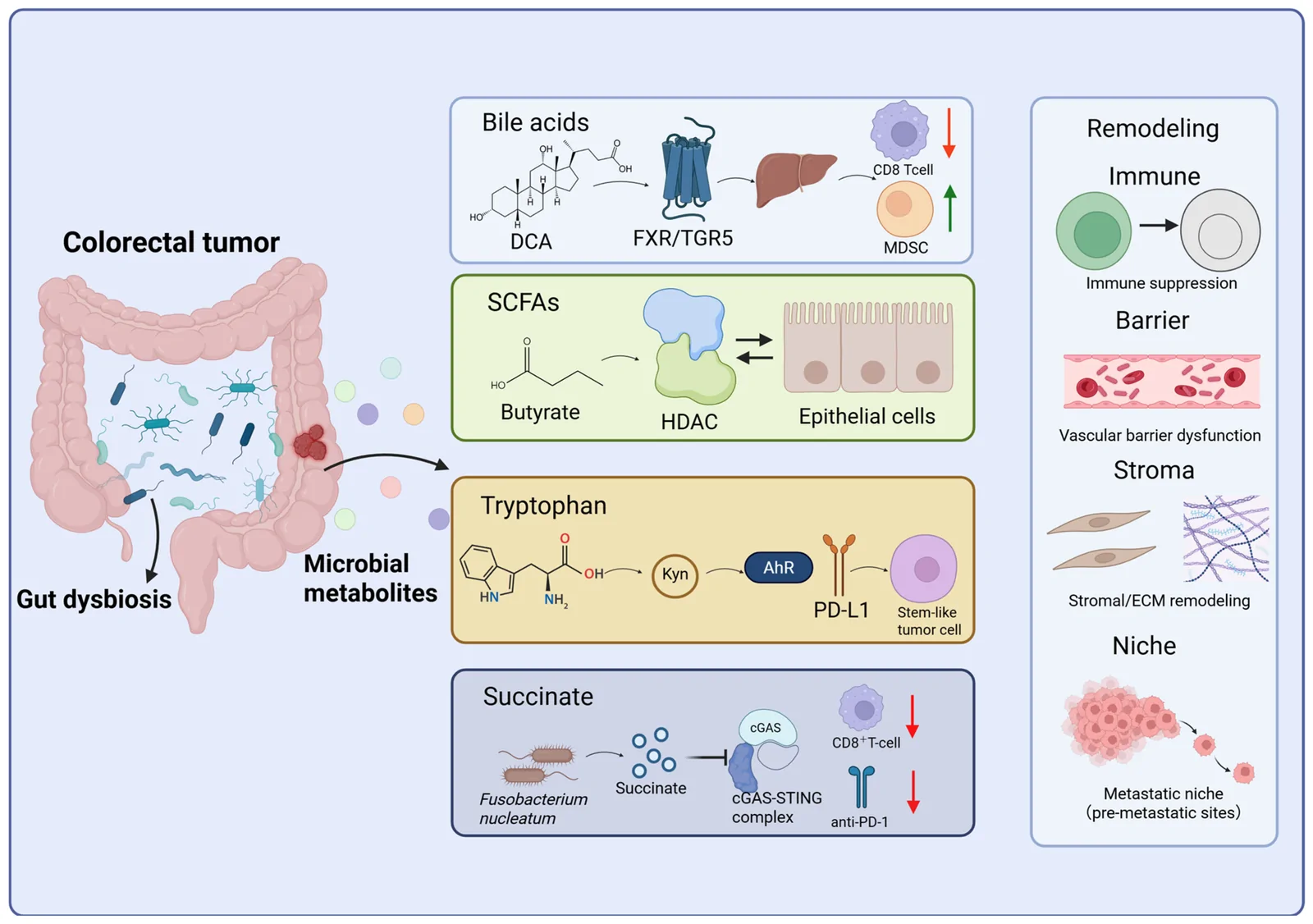
Microbial metabolite axes in colorectal cancer metastasis and therapeutic response. Gut dysbiosis and tumor-associated microbiota can alter microbial metabolite production. Secondary bile acids, short-chain fatty acids, tryptophan-derived metabolites, and *Fusobacterium nucleatum*-derived succinate may influence immune, epithelial, vascular, and stromal remodeling through FXR/TGR5, HDAC-related regulation, Kyn–AhR signaling, and cGAS–STING inhibition. These pathways may affect CD8^+^ T-cell function, MDSC recruitment, PD-L1 expression, barrier integrity, extracellular matrix remodeling, metastatic niche formation, and treatment response. The effects are context-dependent and vary according to metabolite type, dose, host and tumor background, microbial community, metastatic site, and treatment exposure. Abbreviations: AhR, aryl hydrocarbon receptor; DCA, deoxycholic acid; FXR, farnesoid X receptor; HDAC, histone deacetylase; Kyn, kynurenine; MDSC, myeloid-derived suppressor cell; SCFAs, short-chain fatty acids; TGR5, Takeda G protein-coupled receptor 5.

Recent evidence suggests that gut microbiome-derived metabolite axes may influence metastatic susceptibility and organ-specific tumor–microenvironment interactions in CRC by modulating immune, metabolic, barrier-related, and gut–liver signaling pathways. The major microbial metabolite axes and their proposed relationships with microenvironmental remodeling, metastatic niche formation, and treatment response are summarized in Figure 3.

Microbially modified secondary bile acids, such as deoxycholic acid (DCA), have been reported to impair effector CD8^+^ T-cell function and alter antitumor immune activity, thereby creating conditions that may favor tumor progression in selected experimental settings [79]. Across patient specimens, organoids, and mouse models, DCA-related bile acid enrichment was linked to reduced CD8^+^ effector function and accelerated CRC progression, supporting a mechanistic connection among diet, microbial bile acid metabolism, and immune regulation. This concept is consistent with additional preclinical evidence that non-absorbable antibiotic treatment can suppress CRLM in mouse models by altering intestinal microbial DCA metabolism, although these findings should not be generalized as a universal bile acid–metastasis axis [80]. A recent review further summarized evidence that microbial bile acids regulate epithelial, immune, and inflammatory pathways through receptors such as farnesoid X receptor (FXR) and Takeda G protein-coupled receptor 5 (TGR5), providing a receptor-level framework for their possible involvement in metastatic biology. Additional studies suggest that TGR5 activation by microbiota-derived bile acids can recruit myeloid-derived suppressor cells (MDSCs) in the liver and alter the hepatic immune niche, with implications for CRLM progression [81]. A CRLM-focused review also supports the broader relevance of bile acid metabolism to liver metastatic biology, while emphasizing that bile acid effects depend on bile acid species, receptor context, liver immune state, diet, and microbial community composition [82].

Secondly, short-chain fatty acids (SCFAs) are better described as context-dependent microbial metabolites with immunometabolic effects, rather than uniformly protective factors. Among them, butyrate, a histone deacetylase (HDAC) inhibitor and epithelial energy substrate, has shown anti-metastatic activity in selected experimental settings. In a CRLM mouse model, butyrate supplementation improved microbial dysbiosis-related features, reduced intrahepatic immunosuppressive signals, and lowered liver metastatic burden. Earlier animal studies also reported that butyrate interfered with steps related to hepatic dissemination and colonization, findings that are broadly consistent with recent clinical and mechanistic reviews discussing butyrate-producing taxa in CRC progression [83,84]. However, SCFA effects vary by metabolite type, dose, host background, diet, and experimental system. For example, high-dose propionate promoted immune escape through upregulation of the PD-L1/insulin-like growth factor 2 mRNA-binding protein 3(IGF2BP3) axis in vitro and in animal models, indicating that SCFAs should not be generalized as uniformly anti-metastatic metabolites [85]. Future translational applications will therefore need to define dose–response relationships and host-specific factors more precisely.

Thirdly, amino acid metabolism, particularly the tryptophan pathway, has been implicated in metastatic phenotypes in CRC. In this pathway, tryptophan catabolism through tryptophan 2,3-dioxygenase 2 (TDO2) and indoleamine 2,3-dioxygenase 1 (IDO1) generates kynurenine (Kyn), which can activate the aryl hydrocarbon receptor (AhR). AhR signaling has been linked to stemness-associated transcriptional programs that sustain cancer stem cell (CSC)-like populations and to increased PD-L1 expression [86]. In human-derived organoids, xenografts, and liver metastasis models, inhibition of the TDO2–Kyn–AhR pathway or PD-L1 knockout reduced liver metastatic capacity [86]. At the population level, circulating kynurenine/tryptophan (Kyn/Trp) metabolite profiles have been associated with CRC survival outcomes in large multi-cohort analyses, extending the relevance of tryptophan metabolism from experimental models to clinical prognostic associations [87]. In addition, microbial metabolites such as succinate have been linked to immunotherapy response through innate immune sensing pathways. In mouse CRC models, *F. nucleatum*-derived succinate inhibited cGAS–STING–IFN-β signaling, restricted CD8^+^ T-cell infiltration, and reduced anti-PD-1 activity [54,55]. This mechanism provides a bridge between microbial metabolism, innate immune signaling, and adaptive antitumor immunity without implying that succinate alone explains metastatic susceptibility or treatment failure. Taken as a whole, secondary bile acids, SCFAs, and the tryptophan–Kyn–AhR pathway illustrate how microbial metabolism can intersect with immune regulation, epithelial and vascular barrier function, and organ-specific metastatic niches in CRC. Rather than acting as uniformly harmful or protective metabolites, these pathways appear to exert context-dependent effects shaped by diet, microbial community structure, host immunity, tumor site, and treatment exposure. Their translational value may therefore lie in refining metabolic risk stratification and identifying candidate intervention points, including rational combinations of metabolic modulation with chemotherapy or immune checkpoint inhibitors (ICIs), once supported by appropriately designed clinical studies.

### 3.5. Multi-Kingdom Interactions in Metastatic Progression

Beyond bacterial dysbiosis, non-bacterial residents of the gut microbiome—namely fungi, the mycobiome, and viruses, the virome—are increasingly recognized as components of the metastatic ecosystem. Fungal dysbiosis, characterized by an altered Basidiomycota-to-Ascomycota ratio and reduced abundance of *Saccharomyces cerevisiae*, has been reported in mCRC patients [10]. In experimental settings, selected fungi such as *Candida tropicalis* can modulate antitumor immunity by enhancing the suppressive capacity of myeloid-derived suppressor cells (MDSCs). Fungal stimulation induces metabolic reprogramming in MDSCs through pyruvate kinase M2 (PKM2)-dependent aerobic glycolysis, which increases their ability to suppress cytotoxic CD8^+^ T-cell proliferation and may contribute to an immune milieu permissive to tumor progression [9]. Furthermore, multi-kingdom network analyses suggest that fungi and bacteria may interact within shared ecological structures, including mixed biofilms, potentially influencing microbial persistence, host immune exposure, and intestinal barrier integrity [88].

Concurrently, the gut virome has emerged as a non-bacterial component of the microbiome that may influence antitumor immunity and treatment-related phenotypes. Sequencing studies indicate that enteric virome diversity is altered in CRC, and selected viral signatures have been associated with poor survival outcomes [11]. This virome shift is partly driven by bacteriophages, including expansion of Caudovirales. Phages may modulate bacterial community function by acting as vectors for horizontal gene transfer, potentially disseminating virulence-associated or antibiotic-resistance genes among bacterial populations under therapeutic or inflammatory stress [89].

Moreover, phages may translocate across a disrupted gut–vascular barrier and interact with host immune cells. Toll-like receptor 9 (TLR9), for example, recognizes unmethylated CpG motifs in phage DNA. This interaction has been linked to mucosal inflammation, interleukin-6 (IL-6) production in macrophages, and altered adaptive immune surveillance. In experimental settings, gut virome dysbiosis has been associated with reduced CD8^+^ T-cell activity within the tumor microenvironment and diminished response to 5-FU-based chemotherapy [12]. In addition to bacteriophages, eukaryotic oncogenic viruses, such as JC polyomavirus (JCPyV) and Epstein–Barr virus (EBV), have been detected in CRC tissues and have been discussed in relation to epithelial infection, chromosomal instability, and anti-apoptotic signaling [90,91,92,93]. Together, these multi-kingdom interactions among bacteria, fungi, and viruses expand the ecological framework of mCRC metastasis and suggest that non-bacterial microbial communities may contribute to immune, metabolic, and barrier-related remodeling. This area represents an emerging frontier for mechanistic investigation and microbiome-informed intervention, particularly once supported by CRC-specific metastatic models and longitudinal patient cohorts.

## 4. Microbiome-Associated Biomarkers in Metastatic CRC

### 4.1. Microbiome-Associated Biomarkers for CRC Detection and Metastasis Risk

Microbiome dysbiosis in CRC has been investigated as a candidate source of biomarkers for tumor detection and risk assessment [94]. Several fecal microbiome analyses have reported that CRC patients differ from healthy individuals in the relative abundance of taxa such as *Fusobacterium*, *Peptostreptococcus*, *Porphyromonas*, and *Parvimonas*, together with depletion of taxa commonly associated with gut homeostasis [95,96,97,98]. For CRC screening, microbial groups including *Fusobacterium* and *Enterococcus*, as well as selected oral-associated taxa, have been incorporated into fecal metagenomic models for detecting colorectal neoplasia. Several machine-learning models based on fecal metagenomics have shown diagnostic discrimination for early-stage CRC or adenomas, with reported average area under the receiver operating characteristic curve (AUC) values of 0.80–0.85 [99,100]. For example, a study pooling 3741 fecal metagenomes from 18 cohorts identified reproducible microbial features for CRC detection, with a gut microbiome-based prediction model achieving an average AUC of 0.85. These data suggest that microbiome alterations may emerge during the adenoma–carcinoma sequence and could contribute to non-invasive risk assessment when combined with established clinical and screening approaches. Another multi-cohort analysis integrating 1056 samples constructed a random forest model with 11 bacterial features that distinguished intestinal adenomas from healthy controls (AUC = 0.80; independent validation AUC = 0.78), while a 26-feature model distinguished adenomas from CRC with an AUC = 0.89. The major microbiome-associated biomarkers reported in metastatic CRC, together with their clinical contexts, directions of association, proposed implications, and evidence limitations, are summarized in Table 1.

In addition to tumor detection, microbiome composition has also been examined in relation to tumor invasion and metastasis risk. Studies have reported associations between intratumoral microbial diversity and metastatic status in CRC: tumors with higher microbial α-diversity were more frequently observed in patients with distant metastases, and microbial composition showed discriminatory ability between metastatic and non-metastatic cases [106]. A retrospective study by Parajuli et al. inferred microbial profiles from RNA-sequencing data of nearly 900 CRC tumors and found that higher inferred abundance of *B. fragilis* and lower abundance of *F. nucleatum* were associated with shorter metastasis-free survival, whereas metastatic lesions showed enrichment of *E. coli* and other members of the *Enterobacteriaceae* family [106]. Wu et al. further compared the fecal microbiome of 147 CRC patients and reported that lymph node metastasis was associated with higher gut abundance of *Bacteroides plebeius*, together with increased neutrophil-related signals and C-X-C motif chemokine ligand 8 (CXCL8) [30]. Thus, these findings support the potential value of microbial features as adjunctive markers for metastasis risk stratification, but they should be interpreted in relation to tumor site, sample type, host inflammatory state, treatment exposure, and whether microbial enrichment precedes or follows metastatic spread.

### 4.2. Microbiome Composition as a Prognostic Biomarker in mCRC

The composition of tumor-associated and fecal microbiomes in patients with CRC has been associated with survival outcomes, suggesting that microbial compositional features may serve as candidate prognostic biomarkers. In particular, the abundance of *F. nucleatum* in CRC tissues has been widely studied: multiple studies and meta-analyses have consistently pointed out that patients with a high abundance of *F. nucleatum* in their tumors have significantly poorer overall survival (OS) and disease-free survival (DFS). A systematic review by Kim et al. integrated data from 3199 patients and found that high expression of *F. nucleatum* was significantly associated with poorer OS, DFS, and cancer-specific survival (*p* < 0.05) [101]. Further analysis indicated that the abundance of this bacterium is also related to tumor location, microsatellite instability (MSI), and BRAF mutation status. Oral-associated microbial taxa have also been examined in relation to CRC prognosis. A 2025 study of 312 patients with CRC analyzed the salivary microbiome using 16S rRNA sequencing and found that increased abundances of *Neisseria oralis* and *Campylobacter gracilis* were associated with higher risks of disease progression and death (hazard ratio ≈ 2.3 for each), whereas a higher abundance of *Treponema medium* was associated with a lower risk (HR ≈ 0.4) [110]. The study constructed a microbial risk score (MRS), and combining the MRS with clinical factors such as tumor stage improved prognostic discrimination for recurrence and progression, with the C-index of the combined model reaching 0.77.

Furthermore, some studies have compared the relationship between the intratumoral microbiota and postoperative recurrence: in an analysis of stage III CRC, although there was no significant overall difference in the microbial α-diversity between patients who recurred and those who did not, incorporating the microbial composition into the prediction model was significantly better at predicting recurrence than using clinical factors alone (AUC: 0.846 vs. 0.679, *p* = 0.009) [112]. Especially in female patients, the prediction AUC of the model including microbial features was significantly higher than that of the traditional pathological model in the validation cohort. This evidence suggests that intratumoral microbial composition may complement clinicopathological variables in recurrence-risk modeling.

Because CRC progression involves host genetics, metabolism, immunity, and microbial ecology, integrating microbiome data with other omics layers may improve disease subtyping and prognostic stratification [98,113,114].

Recent studies have attempted to combine metagenomic, transcriptomic, metabolomic, immunomic, and genomic data to identify molecular subtypes and survival-associated features. Wang et al. performed an integrated analysis of genomic, transcriptomic, immunomic, and microbiome data from 274 CRC patients and classified tumors into two major subtypes, CS1 and CS2, with distinct prognostic outcomes [115]. A multi-omics scoring model, the microbiome-associated composite multi-omics learning score (MCMLS), showed discriminatory ability for patient survival and potential for stratifying response to immune checkpoint inhibitors (ICIs), although its robustness across populations and treatment settings requires further evaluation. In another direction, models combining fecal metagenomic and metabolic indicators have achieved high AUC values for distinguishing CRC patients from negative controls [116]. International collaborative studies have also integrated multi-kingdom biomarkers from bacteria, fungi, and archaea to define microbial signatures associated with CRC prognosis [88].

Machine-learning approaches have further been applied to microbiome-based risk modeling. Wu et al. constructed random forest (RF) and multilayer perceptron (MLP) models to predict lymph node metastasis, achieving favorable accuracy and AUC in training and validation sets [30]. Logistic regression models incorporating tumor microbiota improved recurrence discrimination in stage III CRC compared with models based on clinical factors alone [112]. The MCMLS model also showed predictive value for survival and immunotherapy response across datasets [115]. Emerging approaches, such as explainable artificial intelligence (XAI), may help identify interpretable microbial features and generate testable biological hypotheses. Overall, these computational models are best viewed as promising components of microbiome-informed risk-stratification research rather than stand-alone tools for clinical management of metastatic CRC.

Despite the growing promise of microbiome-based biomarkers for detection and prognostic assessment in mCRC, their routine clinical implementation remains exploratory. First, gut microbiome composition exhibits substantial geographic and population-level variability; predictive models or microbial panels trained in specific cohorts often show reduced reproducibility when applied to populations with different genetic backgrounds, ethnicities, environmental exposures, dietary patterns, and treatment histories [117]. Microbial composition is also highly susceptible to external influences, including lifestyle changes, antibiotic use, bowel preparation, hospitalization, and systemic therapy, which introduce confounding variables that are difficult to control in real-world clinical settings [118]. Furthermore, the biological meaning of specific microbial signatures remains context-dependent. As illustrated by the driver–passenger framework, enrichment of a given taxon may reflect functional participation in barrier injury, immune or metabolic remodeling, secondary colonization of an altered tumor niche, or ecological selection by hypoxia, necrosis, mucin availability, treatment exposure, or sampling conditions [119]. Large-scale, multicenter, and standardized prospective studies with longitudinal sampling and external validation will therefore be required before microbiome biomarkers can be reliably evaluated for clinical integration in mCRC.

## 5. Microbiome-Based Interventions and Clinical Translation

Microbiome-informed therapeutic strategies in mCRC span several treatment contexts, including chemotherapy, radiotherapy, immunotherapy, and targeted therapy. To provide an overview of these translational directions, Table 2 summarizes representative microbiome-associated interventions and treatment-related mechanisms in mCRC, including probiotic supplementation, selective antimicrobial or bacteriophage-based targeting, microbial drug-metabolizing enzymes, FMT, microbial metabolite pathways, and microbiome-associated modulation of treatment response and toxicity.

### 5.1. Microbiome and Chemotherapy in mCRC

The microbiome has been linked to variability in chemotherapy response, toxicity, and drug metabolism in mCRC. Specific microbial taxa have been associated with treatment-related phenotypes in selected clinical and experimental studies. For instance, *F. nucleatum* has been reported to reduce chemotherapy sensitivity through activation of the Toll-like receptor 4 (TLR4)–myeloid differentiation primary response 88 (MYD88) signaling axis, promotion of autophagy in tumor cells, and attenuation of the cytotoxic effects of fluoropyrimidines such as 5-FU and platinum-based agents such as oxaliplatin [63]. Conversely, enrichment of taxa such as *Roseburia faecis* and *B. fragilis* within the tumor microenvironment has been correlated with more favorable responses to fluoropyrimidine-based chemotherapy in selected cohorts [107,108]. Several microbial species can also modulate drug metabolism via enzymatic mechanisms; for example, microbial β-glucuronidases can deconjugate SN-38G—the glucuronidated inactive hepatic metabolite of irinotecan—back to SN-38 within the gastrointestinal tract, thereby contributing to localized toxicity. Additionally, microbial dihydropyrimidine dehydrogenase (DPD) activity, particularly from *E. coli*, can degrade 5-FU and may attenuate its anticancer efficacy [61,123,124]. Beyond the bacterial microbiome, emerging evidence from gut virome studies suggests that viral dysbiosis may alter antitumor immunity and 5-FU response in experimental settings [12].

Selected microbial taxa and probiotic formulations have been examined for their potential to support chemotherapy tolerability or response. Probiotic formulations containing selected lactic acid bacteria or bifidobacteria have been investigated for the mitigation of chemotherapy-associated gastrointestinal toxicity, although the available evidence remains regimen- and population-specific [120,121]. Similarly, *Roseburia* spp. produce butyrate, a short-chain fatty acid with immunomodulatory properties, and have been discussed in relation to antitumor immune activity during chemotherapy [108,138,139]. Elevated relative abundance of *B. fragilis* and *R. faecis* has been associated with more favorable chemotherapeutic outcomes in patients with gastrointestinal malignancies [108]. Microbial-derived metabolites such as 3-indoleacetic acid (3-IAA), synthesized by selected gut bacteria, have also been reported to enhance chemotherapy-associated antitumor effects in vivo, although much of this evidence remains cross-cancer or preclinical rather than mCRC-specific [122]. Additional systematic and mechanistic reviews support the broader concept that gut microbiota may influence chemotherapy response, efficacy, and toxicity through microbial metabolism, immune modulation, and drug-metabolizing enzymes [107,122,140].

Therapeutic manipulation of the microbiome has thus emerged as a promising adjunctive strategy. Probiotic formulations, including *Lacticaseibacillus rhamnosus* GG, *L. casei*, and *B. breve*, have been studied for their ability to reduce chemotherapy-induced gastrointestinal toxicity. In murine models, a multi-strain probiotic cocktail containing *Lactobacillus*, *Bifidobacterium*, and *Streptococcus* attenuated 5-FU–induced intestinal injury [121]. A clinical study also reported that *L. rhamnosus* GG reduced the incidence and severity of 5-FU–associated diarrhea in CRC patients [120]. Beyond probiotic supplementation, selective antimicrobial strategies have also been explored in preclinical settings. In xenograft models enriched for *F. nucleatum*, metronidazole treatment reduced tumor progression and *Fusobacterium* burden, supporting further investigation of selective microbial targeting as a chemotherapy-adjunctive strategy [17]. Finally, although FMT has been predominantly studied in the context of immunotherapy, its potential application during chemotherapy is being considered. By restoring microbial diversity and increasing taxa capable of producing mucosal-supportive short-chain fatty acids, FMT could theoretically help mitigate chemotherapy-induced mucosal injury, but its role in chemotherapy-treated mCRC remains to be defined in controlled clinical studies. Because chemotherapy, supportive medications, antibiotics, nutritional interventions, and hospitalization can reshape gut microbial ecology, future studies should distinguish pretreatment predictive signatures from treatment-emergent microbial changes through longitudinal sampling during chemotherapy.

### 5.2. Microbiome and Immunotherapy in mCRC

Immune checkpoint inhibitors (ICIs) have limited efficacy in CRC except for mismatch repair-deficient or microsatellite instability-high (MSI-high) tumors, and growing evidence indicates that gut microbiome composition is associated with variability in ICI response across cancer types. Several responder-associated taxa, including *Akkermansia muciniphila*, *B. fragilis*, *Bifidobacterium* spp., *Faecalibacterium prausnitzii*, *Eubacterium limosum*, and *Alistipes shahii*, have been linked to improved responses to PD-1/PD-L1 or cytotoxic T-lymphocyte-associated protein 4 (CTLA-4) blockade in selected studies [133,141,142]. Mechanistically, these taxa have been proposed to support antitumor immunity through dendritic-cell activation, cytotoxic T-cell function, and immune-metabolic signaling. At the same time, taxa classically linked to CRC progression may show treatment-context-dependent effects. *F. nucleatum* has often been associated with CRC progression, immune suppression, and poor therapeutic response [102,103,104], yet in a mouse model it enhanced anti–PD-L1 activity by activating STING–nuclear factor-κB(NFκB) signaling and increasing PD-L1 expression in tumor cells [105]. Similarly, ETBF induced colonic tumors in mice but also increased sensitivity to anti–PD-L1 treatment in a specific experimental context [109]. These examples illustrate that taxon-level associations with ICI response should be interpreted according to tumor model, baseline immune state, microbial community structure, and treatment regimen, rather than as fixed beneficial or harmful properties of individual microbes.

Key immunomodulatory mechanisms include microbial metabolites and immune signaling. High-fiber diets and prebiotics can increase fermentable-substrate availability and support the production of short-chain fatty acids (SCFAs), while bacterial cyclic diadenosine monophosphate (c-di-AMP) can activate STING–type I interferon signaling [128,129,130,132,143,144]. In selected settings, SCFAs such as butyrate and propionate have been linked to CD8^+^ T-cell activity, and c-di-AMP-mediated innate immune signaling has been associated with enhanced antitumor immune responses [130]. Conversely, fiber deprivation may promote mucus barrier erosion by mucin-degrading bacteria such as *A. muciniphila* and increase colitis susceptibility, highlighting the context-dependent nature of diet–microbiome–immunity interactions [145]. Strategies based on these observations include prebiotics, such as inulin and pectin, to alter fermentable-substrate availability [131]. In an open-label, single-arm phase II trial of refractory microsatellite-stable (MSS) mCRC, FMT combined with tislelizumab and fruquintinib achieved a 20% response rate and 95% disease-control rate [23]. Responders were enriched in *Proteobacteria* and *Lachnospiraceae*, whereas non-responders had higher *Actinobacteria* and *Bifidobacterium*. Together with early FMT studies in immunotherapy-refractory melanoma, these findings support the feasibility of microbiome-informed immunotherapy modulation across selected cancer contexts, but they do not establish that microbiome transfer alone can overcome ICI resistance in MSS mCRC [146,147].

For FMT-containing immunotherapy regimens, longitudinal sampling before FMT, during hospitalization or treatment, and after therapy will be necessary to determine whether response-associated microbial shifts reflect donor engraftment, treatment-induced ecological remodeling, antibiotic or dietary exposures, or tumor response itself.

Probiotics and next-generation microbial therapeutics are also under study. *A. muciniphila* or its derivatives, including pasteurized bacteria or outer-membrane proteins, have been reported to enhance PD-1/PD-L1 blockade responses and promote a more inflamed tumor immune phenotype in experimental settings [134]. Engineered microbial therapeutics, such as *Lactococcus lactis* secreting tumor-targeted nanobodies against PD-L1/CTLA-4, have shown proof-of-concept in preclinical MSS CRC models [135]. These studies support microbiome-informed immunotherapy modulation as an emerging translational direction, while patient selection, safety, dosing, durability, microbial baseline state, and regimen-specific effects remain to be defined.

### 5.3. Microbiome and Radiotherapy in mCRC

Gut microbiome composition has also been investigated as a modifier of radiotherapy response and toxicity in CRC. Radiotherapy can substantially alter gut microbial communities, including reductions in microbial load and enrichment of inflammation-associated taxa, which may contribute to gastrointestinal (GI) toxicity such as diarrhea, mucositis, and inflammation [148]. Specific microbial taxa have also been examined in relation to tumor radiosensitivity. *Roseburia intestinalis*, a butyrate-producing taxon, was reported to increase radiation-induced CRC cell death in experimental settings. In mouse CRC models, *R. intestinalis* promoted cancer-cell autophagy through butyrate signaling involving olfactory receptor 51E1 (OR51E1) and Ras-related protein Ral-B (RALB), thereby increasing radiotherapy sensitivity [111]. This finding is consistent with broader evidence that gut microbiota and microbial metabolites may influence radiotherapy response and radiation-induced GI injury, although CRC-specific clinical validation remains limited [149]. ETBF has been linked to inflammatory and STAT3-related signaling in experimental CRC models. Separately, activation of the Janus kinase 2 (JAK2)–STAT3–cyclin D2 (CCND2) axis has been associated with cancer stem-cell persistence and radioresistance. Whether ETBF directly contributes to CRC radioresistance through this pathway remains to be established [42,150].

In mouse models, FMT after irradiation increased production of indole-3-propionic acid (IPA), a bacterial tryptophan metabolite, through expansion of *Clostridium sporogenes*; IPA was reported to preserve intestinal stem-cell function and reduce radiation-induced intestinal injury [136,137]. This microbiome–metabolite axis provides mechanistic support for radiation-injury mitigation in experimental models, but it should not be taken as direct evidence that IPA-based interventions improve CRC radiotherapy outcomes [137]. These findings frame two radiotherapy-related research directions: normal-tissue protection through microbiome-associated barrier and metabolic support, and tumor radiosensitization through selected microbial or metabolic pathways. However, the relevance of targeting *R. intestinalis*, IPA-producing pathways, or antibiotic-based microbiome modulation in CRC radiotherapy remains to be established in clinically relevant studies.

### 5.4. Microbiome and Targeted Therapy in mCRC

Targeted agents in mCRC include epidermal growth factor receptor (EGFR)-targeted antibodies, such as cetuximab and panitumumab; vascular endothelial growth factor (VEGF)-targeted therapy, such as bevacizumab; and multikinase inhibitors, such as regorafenib and fruquintinib. Emerging evidence suggests that gut microbiome composition may be associated with the efficacy and toxicity of these therapies. In one study of first-line EGFR/VEGF therapy in mCRC patients, microbiome composition predicted response: patients with partial response (PR) harbored more *F. nucleatum* and certain *Prevotella* species in their gut, whereas patients with progressive disease (PD) showed enrichment of *Klebsiella quasipneumoniae*, *Lactobacillus mucosae*, various *Bifidobacterium* spp., and *Veillonella* spp. [13]. This indicates that baseline microbial composition may be associated with treatment response; however, whether *F. nucleatum* directly modifies the efficacy of EGFR- or VEGF-targeted therapy remains unclear.

Notably, tumor-resident *Bacillus* spp. in CRC tissues were associated with longer OS and PFS in the cited cohort under cetuximab. In a cohort of 65 mCRC patients, those with high *Bacillus* (and *Coprococcus*) expression had significantly longer overall survival on cetuximab (median 34.1 vs. 19.4 months) and progression-free survival (12.6 vs. 6.2 months) [126]. Conversely, high *Streptococcus* levels predicted worse survival. Preclinical evidence indicates that *Bacillus polyfermenticus* can inhibit erythroblastic oncogene B receptor 2/3 (ErbB2/ErbB3)-related signaling in vitro; however, whether this mechanism contributes to cetuximab response in patients with mCRC remains unknown [127].

Bacterial metabolism can also affect small-molecule kinase inhibitors. For example, certain gut β-glucuronidases reactivate the glucuronidated form of regorafenib in the intestine, raising local drug concentration and toxicity [125]. Studies in mice show that blocking these enzymes reduces GI side effects of regorafenib. Moreover, microbial products like deoxycholic acid can transactivate EGFR, whereas ursodeoxycholic acid antagonizes human epidermal growth factor receptor 2 (HER2) signaling [151], hinting that the bile acid pool (shaped by microbiome) might influence responsiveness to EGFR/HER2 inhibitors. While data in CRC patients remain sparse, these concepts underscore that the microbiome–metabolome can modulate targeted drug pathways.

Collectively, available evidence suggests that gut and tumor-associated microbial features may be associated with treatment efficacy and toxicity across several therapeutic modalities in mCRC. Selected taxa have been linked to differential therapeutic responses in specific clinical or experimental settings, potentially through immune regulation, microbial drug metabolism, metabolite-mediated signaling, and modulation of epithelial barrier integrity. Although antibiotics, probiotics or synbiotics, fecal microbiota transplantation, and dietary interventions are being investigated as microbiome-modulating approaches, current evidence does not establish their routine clinical value in mCRC or demonstrate that microbiome modulation itself improves oncological outcomes. Prospective, longitudinal, and adequately controlled studies are therefore required to validate predictive microbial signatures and determine whether microbiome-directed interventions can safely benefit selected patients.

## 6. Current Challenges and Critical Controversies in mCRC Microbiome Research and Translation

Although microbiome research has reshaped the biological framework of mCRC metastasis and treatment response, the current evidence base still requires careful interpretation. The most fundamental controversy is whether metastasis-associated microorganisms are causal drivers of progression or secondary colonizers of an already remodeled tumor ecosystem. Fecal, tumor-tissue, multi-kingdom, and machine-learning studies have identified microbial signatures with prognostic or predictive value [30,88,106,112,115], but many of these associations are derived from retrospective or cross-sectional cohorts and therefore cannot establish temporal directionality. Mechanistic findings support causal roles for selected taxa, such as the persistence of *Fusobacterium* in paired primary and metastatic lesions and bacterial dissemination after gut–vascular barrier disruption [17,78]. However, these examples should not be overgeneralized to all dysbiotic microorganisms. As proposed in the driver–passenger framework, some bacteria may functionally participate in barrier injury, immune remodeling, or metabolic reprogramming, whereas others may preferentially colonize metastatic lesions after the tissue ecosystem has already been remodeled. Beyond immune and metabolic mechanisms, microbial enrichment in metastatic tissues may also reflect physical and ecological selection. Oxygen availability is a major determinant of microbial community structure: intraluminal oxygen gradients influence the spatial organization of intestinal microbiota, and epithelial metabolic disruption can increase oxygen availability and favor expansion of facultative anaerobes such as *Enterobacteriaceae* [152,153]. Inflammatory remodeling can further alter microbial nutrient availability; for example, host-derived nitrate generated during inflammation can support the growth of *E. coli* in inflamed gut environments [154]. Mucin availability represents another ecological determinant, because mucin glycans provide nutrient sources and adhesion-related substrates for mucin-adapted bacteria [155,156]. In metastatic tissues, additional features such as hypoxia, necrotic debris, altered tissue perfusion, local pH, ascites, and treatment-induced epithelial or vascular injury may create permissive microhabitats for microbial persistence or detection [157]. These ecological constraints may differ substantially across liver, lung, lymphatic, and peritoneal metastases and may also be modified by diet, bowel preparation, antibiotics, surgery, chemotherapy, targeted therapy, and hospitalization [158,159,160]. Therefore, enrichment of a given taxon in metastatic tissue should not be interpreted solely as evidence of oncogenic or pro-metastatic activity; it may also reflect ecological filtering, passive colonization, or treatment-shaped niche remodeling.

A second challenge lies in reproducibility and methodological standardization. Microbiome composition is strongly shaped by geography, ethnicity, diet, lifestyle, antibiotic exposure, bowel preparation, sampling site, sequencing platform, and bioinformatic pipeline [117,118]. These confounders are particularly relevant in mCRC, because patients often undergo repeated chemotherapy, targeted therapy, surgery, hospitalization, nutritional intervention, and antibiotic treatment, all of which can reshape the gut ecosystem independently of tumor biology. Importantly, most available studies rely on baseline or single time-point fecal or tissue microbiome profiling and do not systematically capture the in-hospital or treatment-period microbiome, where many clinically relevant exposures occur. This limitation makes it difficult to determine whether therapy-associated microbial signatures precede treatment response, emerge as a consequence of treatment, or reflect hospitalization-related factors such as antibiotics, diet modification, parenteral or enteral nutrition, perioperative stress, infection, and supportive medications. Longitudinal sampling before, during, and after treatment, ideally paired with detailed annotation of hospital exposures, will therefore be necessary to distinguish predictive microbial features from treatment-shaped ecological changes. Moreover, fecal microbiome profiles may not fully represent tumor-resident or metastatic-site microbiota, whereas tissue-based microbial analyses are vulnerable to low-biomass contamination, reagent-derived signals, cross-sample contamination, batch effects, and sequencing or taxonomic-classification artifacts. These issues are especially important for metastatic lesions, peritoneal fluid, ascites, and small tissue biopsies, where low microbial biomass can make contaminant taxa appear biologically meaningful. Therefore, microbial panels trained in one cohort may lose accuracy when applied to populations with different genetic backgrounds, treatment histories, dietary patterns, metastatic sites, or sample-processing workflows. Future studies should adopt standardized protocols for sample collection, storage, DNA extraction, sequencing depth, negative extraction controls, reagent blanks, batch-aware statistical analysis, and bioinformatic contaminant detection, while moving beyond genus-level correlations toward strain-level, functional, spatial, quantitative, and metabolomic validation.

Therapeutic manipulation of the microbiome is also controversial because the biological effects of individual taxa and metabolites are highly context-dependent. *F. nucleatum* has often been associated with tumor progression, chemoresistance, immune suppression, and poorer therapeutic response in CRC [63,102,103,104], yet experimental evidence indicates that it may enhance anti-PD-L1 efficacy under specific immunological conditions [105]. Similarly, ETBF can induce inflammatory and tumor-promoting signaling in experimental settings, but bacterial-driven immune activation in certain contexts may increase sensitivity to immune checkpoint blockade [42,109]. Microbial metabolites show comparable duality: short-chain fatty acids (SCFAs), including butyrate, are often described as immunomodulatory metabolites with barrier-supportive or antitumor effects, whereas high-dose propionate may promote immune escape through the PD-L1/IGF2BP3 axis [83,84,85]. These apparently conflicting observations argue against a simplistic division between “beneficial” and “harmful” microbes. Antibiotics, probiotics, prebiotics, FMT, phage therapy, and engineered live biotherapeutics should therefore be developed as precision interventions guided by baseline microbiome structure, tumor genotype, immune status, metastatic site, and treatment modality rather than as uniform adjuncts for all mCRC patients.

Another important limitation is that the strength of evidence varies substantially across microbiome findings. Some microbial associations are supported by multi-cohort analyses, meta-analyses, or cross-cohort validation, whereas others rely on single-center cohorts, small metastatic-tissue datasets, animal models, or individual mechanistic studies. Therefore, findings from one experimental system or one patient cohort should not be generalized to all mCRC settings without considering tumor site, sample type, sequencing platform, treatment exposure, host background, and microbial community context. Cross-study discrepancies should also be expected, because the same taxon may show different associations depending on whether fecal samples, primary tumor tissue, metastatic lesions, or post-treatment specimens are analyzed. Accordingly, throughout this review, single-study findings should be interpreted as mechanistic examples or hypothesis-generating observations unless they have been reproduced across independent cohorts or validated in longitudinal and functionally annotated studies.

Finally, clinical translation remains constrained by the early stage of interventional evidence. Findings such as a metronidazole-mediated reduction in *Fusobacterium*-associated tumor burden, probiotic mitigation of chemotherapy-related diarrhea, FMT combined with immunotherapy-containing regimens in refractory MSS mCRC, and engineered probiotics delivering checkpoint-blockade nanobodies provide important proof-of-concept for microbiome-informed therapeutic modulation [17,23,120,121,135]. Nevertheless, many studies remain preclinical, small-scale, single-arm, or exploratory, and few have demonstrated durable improvements in PFS, OS, or quality of life in randomized settings. Before microbiome-based strategies can be integrated into routine mCRC care, future trials should incorporate longitudinal multi-omics profiling, predefined microbial and metabolic endpoints, donor and product standardization, safety surveillance for live biotherapeutics, and stratification by microsatellite instability/mismatch repair (MSI/MMR) status, RAS/BRAF genotype, metastatic organ involvement, and prior antibiotic exposure. Such a framework will be essential to distinguish microbial features with functional relevance from correlative biomarkers and to define where microbiome-informed strategies can be safely and effectively integrated into mCRC management.

## 7. Conclusions

Current evidence supports a shift from a predominantly tumor-centric view of mCRC toward a more integrated framework that incorporates the gut and tumor-associated microbiome as part of the metastatic ecosystem. By organizing the available evidence around liver, lung, lymphatic, and peritoneal metastatic niches and connecting these patterns with barrier dysfunction, microbial translocation, immune–stromal remodeling, microbial metabolism, methodological limitations, and therapeutic translation, this review provides a framework that complements previous reviews focused primarily on CRC tumorigenesis, general immune modulation, or treatment response. Clinically, metastasis-associated microbial signatures are being evaluated as adjunctive biomarker candidates for risk stratification and prognostic assessment alongside established clinicopathological and molecular markers. Microbiome-targeted approaches, including fecal microbiome transplantation, engineered probiotics, bacteriophage-based strategies, dietary modulation, and selective microbial targeting, are also being explored as candidate adjuncts that may influence microbial ecology, immune tone, treatment toxicity, or therapeutic response in selected settings. However, their translation into routine mCRC care remains constrained by unresolved controversies, including causal attribution, inter-cohort variability, methodological inconsistency, and the context-dependent effects of specific microbes and metabolites. Future research should integrate longitudinal multi-omics profiling with clinically relevant preclinical models to resolve strain- and function-specific mechanisms. Ultimately, well-designed randomized controlled trials will be essential to validate microbiome-informed strategies and determine their impact on treatment response and long-term outcomes in mCRC.

## Figures and Tables

**Table 1:** Microbiome-Associated Biomarkers for mCRC.

Biomarker/Taxon/Gene	Metastatic/Clinical Context	Reported Context	Main Implication	Refs.
*Fusobacterium nucleatum*	CRLM, distant metastasis, prognosis, ICI response	Enriched in several metastatic or poor-prognosis settings; higher abundance linked to shorter PFS/OS, while ICI associations were context-dependent.	Higher tumor burden, immune suppression, shorter PFS/OS; may persist from primary tumor to liver metastasis	[17,19,20,21,22,55,101,102,103,104,105]
*Fusobacterium* spp.	Lung metastasis	Enriched in fecal or tumor profiles of patients with lung metastasis.	Enriched in patients with lung metastasis	[6]
	Mesenteric lymph node involvement	Enriched in node-positive CRC and associated with altered tumor immune infiltration.	Enriched in node-positive cases; associated with tumor immune infiltration	[32]
*Stenotrophomonas maltophilia*	CRC lung metastasis	Higher abundance associated with recurrence, mortality, vascular invasion, and lower 2-year survival.	Higher recurrence, mortality, vascular invasion, and lower 2-year survival; concordant abundance between primary and lung metastasis	[5,6]
*Bifidobacterium*	Lung metastasis	More abundant in non-metastatic patients in the cited cohort.	More abundant in non-metastatic patients; potential protective commensal signal	[6]
*Bacteroides fragilis*	Metastatic CRC, CRLM, chemotherapy response	Enriched in metastatic CRC; treatment associations vary across cohorts and experimental models.	Enriched in metastatic CRC; ETBF linked to immune modulation; some datasets associate *B. fragilis* with better fluoropyrimidine response	[15,41,58,106,107,108]
Enterotoxigenic *B. fragilis*/BFT	CRLM, immune evasion, ICI sensitivity in mouse models	Linked to inflammatory immune remodeling and CRLM; anti-PD-L1 sensitivity reported in a specific mouse model.	IL-6/IL-1β/IL-23 inflammation, immunosuppressive macrophages; increased anti-PD-L1 sensitivity in one mouse model	[41,42,109]
*Escherichia coli*/*Enterobacteriaceae*	Distant metastasis, CRLM, chemotherapy resistance	Enriched in metastatic lesions; CRLM-resident *E. coli* linked to M2-like macrophage polarization and 5-FU resistance.	Metastases enriched with *E. coli*/Enterobacteriaceae; CRLM-resident *E. coli* lactate promotes M2 macrophage polarization and 5-FU resistance	[24,106]
pks^+^ *E. coli*/colibactin	CRC progression, immune suppression, chemoresistance	Linked to genotoxicity, immunosuppressive phenotypes, and treatment resistance in experimental studies.	DNA damage, suppressive myeloid/Th17 milieu, T-cell dysfunction, lipid overload, ICI/chemo resistance	[47,48,49,50,59]
*Bacteroides plebeius*	Lymph node metastasis	Enriched in LNM and associated with CXCL8-related neutrophil infiltration.	Enriched in LNM; associated with neutrophil infiltration and CXCL8 upregulation	[30]
*Peptostreptococcus anaerobius*	Immune evasion, EMT, oxaliplatin resistance	Linked to MDSC recruitment, EMT, and reduced oxaliplatin sensitivity in experimental models.	MDSC recruitment, IL-23/STAT3 activation, EMT and chemotherapy resistance	[45,46]
*Prevotella*, *Selenomonas*, *Peptostreptococcus*, *Porphyromonas*	Lung metastasis prognosis	Higher abundance associated with shorter survival; *Selenomonas* showed the strongest adverse signal.	Overall survival < 24 months; *Selenomonas* independent adverse factor	[6]
*Sphingomonas*	Lung metastasis prognosis	Presence tended to associate with longer survival in the cited cohort.	Tended to associate with longer survival	[6]
*Clostridia*, *Gammaproteobacteria*	Peritoneal metastasis/malignant ascites	Enrichment trends observed in PM/malignant ascites cohorts.	Enrichment trends in patients with PM/malignant ascites	[34]
*Fusobacterium*, *Bacteroides*	CRC peritoneal metastasis	Increased in CRC-PM tissue and associated with immune and mesothelial remodeling.	Increased within tumor microenvironment; linked to neutrophil remodeling and mesothelial transition	[35]
*Neisseria oralis*, *Campylobacter gracilis*	CRC prognosis	Higher abundance associated with increased risks of progression and death.	Higher abundance associated with worse prognosis	[110]
*Treponema medium*	CRC prognosis	Higher abundance associated with lower progression/death risk in the cited cohort.	Higher abundance associated with reduced progression/death risk	[110]
Butyrate/butyrate-producing taxa	CRLM, radioresponse	Butyrate associated with lower liver metastatic burden; *R. intestinalis* increased radiosensitivity in experimental models.	Associated with a lower liver metastatic burden; *Roseburia intestinalis* sensitizes CRC to radiotherapy	[83,84,111]

Reported associations summarize the direction and context of findings in the cited studies and should not be interpreted as evidence of causality. The reported relationships may vary according to sample type, cohort characteristics, disease stage, metastatic site, treatment exposure, sequencing or analytical method, and experimental model. Taxa are presented at the taxonomic resolution reported in the original studies; “spp.” denotes multiple species within a genus. Abbreviations: BFT, *Bacteroides fragilis* toxin; CRC, colorectal cancer; CRLM, colorectal liver metastasis; CXCL8, C-X-C motif chemokine ligand 8; EMT, epithelial–mesenchymal transition; ETBF, enterotoxigenic *Bacteroides fragilis*; 5-FU, 5-fluorouracil; ICI, immune checkpoint inhibitor; IL, interleukin; LNM, lymph node metastasis; MDSC, myeloid-derived suppressor cell; OS, overall survival; PD-L1, programmed death-ligand 1; PFS, progression-free survival; PM, peritoneal metastasis; STAT3, signal transducer and activator of transcription 3; Th17, T helper 17 cell.

**Table 2:** Representative Microbiome-Associated Therapeutic Strategies and Treatment-Response Mechanisms in mCRC.

Strategy/Microbial Feature	Target/Microbiome Mechanism	Disease/Treatment Context	Main Finding	Refs.
Chemotherapy				
*Lacticaseibacillus rhamnosus* GG supplementation	Probiotic prevention of chemotherapy-associated diarrhea	CRC receiving 5-FU-based chemotherapy	Reduced incidence/severity of chemotherapy-associated diarrhea	[120]
Probiotic mixture DM#1	*Lactobacillus*/*Bifidobacterium*/*Streptococcus*-based mucosal protection	5-FU-induced intestinal mucositis	Ameliorated 5-FU-induced mucositis and dysbiosis in rats	[121]
Metronidazole targeting *F. nucleatum*	Selective depletion of *Fusobacterium*-associated chemoresistance/metastasis	CRC xenograft/metastatic models	Reduced tumor growth and *Fusobacterium* burden; supports antibiotic targeting of tumor-associated bacteria	[17]
Phage VA7 targeting *B. fragilis*	Selective bacterial depletion	CRC chemoresistance	Depletion of *B. fragilis* restored chemosensitivity to 5-FU-based treatment	[58]
Inhibition of RIG-I lactylation/targeting *E. coli*–lactate axis	Reversal of macrophage M2 polarization and 5-FU resistance	CRLM	Restored immune balance and reversed 5-FU resistance in CRLM models	[24]
Microbiota-derived 3-IAA	Potentiation of chemotherapy efficacy	Chemotherapy response; cited as cross-cancer mechanistic support	3-IAA enhanced antitumor chemotherapy effects in vivo	[122]
Microbial DPD/β-glucuronidase activity	Microbial drug metabolism	5-FU degradation; irinotecan toxicity	*E. coli* DPD may degrade 5-FU; β-glucuronidases reactivate SN-38G and contribute to irinotecan GI toxicity	[61,123,124]
Targeted Therapy				
Inhibition of gut microbial β-glucuronidases	Reduce intestinal reactivation of regorafenib glucuronide	Regorafenib toxicity	Reduced regorafenib-induced gastrointestinal toxicity in mouse models	[125]
*Bacillus* abundance/potential probiotic modulation	Possible EGFR/ErbB pathway modulation and cetuximab benefit	Cetuximab-treated mCRC	High tissue *Bacillus*/*Coprococcus* associated with longer OS/PFS under cetuximab; *Bacillus polyfermenticus* inhibits ErbB2/ErbB3 in vitro	[126,127]
First-line chemotherapy/targeted therapy microbiome profiling	Response-associated taxa, including *F. nucleatum*, *Prevotella*, *Klebsiella*, *Lactobacillus*, *Bifidobacterium*, *Veillonella*	mCRC chemotherapy + EGFR/VEGF-targeted therapy	Microbiota composition differed between PR and PD; response prediction rather than direct intervention	[13]
Immunotherapy				
FMT + tislelizumab + fruquintinib	Transfer of responder-like gut microbiota	Refractory MSS mCRC	ORR 20%, disease-control rate 95%, median PFS 9.6 months; responders enriched for Proteobacteria and Lachnospiraceae; non-responders enriched for Actinobacteria/*Bifidobacterium*	[23]
Regorafenib + toripalimab with microbiome profiling	Baseline gut microbiota as response modifier	mCRC, especially liver metastasis subgroup	ORR 15.2% overall; only 8.7% in liver metastasis; high pretreatment fecal *Fusobacterium* associated with shorter PFS	[22]
High-fiber/prebiotic approaches, including inulin gel	Increase beneficial taxa, SCFA/c-di-AMP signaling, systemic antitumor immunity	ICI response/tumor immunity	Enhanced systemic antitumor immunity via gut microbiome modulation	[128,129,130,131]
Natural polyphenol microbiota modulation	Circumvention of anti-PD-1 resistance via microbiota effects	ICI resistance	Improved antitumor activity and overcame anti-PD-1 resistance through gut microbiota changes	[132]
*Akkermansia muciniphila*/derivatives	Conversion of “cold” tumors into “hot” immune microenvironment	PD-1/PD-L1 blockade	Potentiated ICI response, but context-dependent pathobiont concerns remain	[133,134]
Engineered *Lactococcus lactis* delivering PD-L1/CTLA-4 nanobodies	Local tumor delivery of checkpoint blockade	Preclinical MSS CRC model	Proof-of-concept for engineered probiotics as local immunotherapy delivery platform	[135]
Radiotherapy				
FMT after irradiation	Restoration of protective microbial metabolites, especially IPA	Radiation-induced intestinal injury	Protected against radiation-induced toxicity; increased IPA-producing microbial functions	[136,137]
Indole-3-propionic acid/*Clostridium sporogenes* axis	Microbial tryptophan metabolite protects intestinal stem cells	Radiation toxicity	IPA protected intestinal stem cells and alleviated acute radiation injury	[137]
*Roseburia intestinalis* supplementation/enrichment	Butyrate–OR51E1–RALB axis	CRC radiotherapy	Sensitized CRC to radiotherapy by promoting radiation-induced cancer cell autophagy	[111]

The table includes clinical associations, exploratory clinical interventions, preclinical therapeutic strategies, and mechanistic studies, which represent different levels of evidence. Findings from animal models, in vitro systems, or non-CRC cancer models should not be directly extrapolated to clinical mCRC. For combination regimens, the reported clinical outcomes cannot be attributed solely to the microbiome-directed component. The disease, treatment, and experimental contexts of each finding are specified in the corresponding columns. Abbreviations: 3-IAA, 3-indoleacetic acid; 5-FU, 5-fluorouracil; c-di-AMP, cyclic diadenosine monophosphate; CRC, colorectal cancer; CRLM, colorectal liver metastasis; CTLA-4, cytotoxic T-lymphocyte-associated protein 4; DPD, dihydropyrimidine dehydrogenase; EGFR, epidermal growth factor receptor; ErbB2/ErbB3, erythroblastic oncogene B receptor 2/3; FMT, fecal microbiota transplantation; GI, gastrointestinal; ICI, immune checkpoint inhibitor; IPA, indole-3-propionic acid; mCRC, metastatic colorectal cancer; MSS, microsatellite-stable; OR51E1, olfactory receptor 51E1; ORR, objective response rate; OS, overall survival; PD, progressive disease; PD-1, programmed cell death protein 1; PD-L1, programmed death-ligand 1; PFS, progression-free survival; PR, partial response; RALB, Ras-related protein Ral-B; RIG-I, retinoic acid-inducible gene I; SCFA, short-chain fatty acid; SN-38G, glucuronidated SN-38; VEGF, vascular endothelial growth factor.

## Data Availability

No new data were created or analyzed in this study. Data sharing is not applicable to this article.
